# A miRNA/CXCR4 signaling axis impairs monopoiesis and angiogenesis in diabetic critical limb ischemia

**DOI:** 10.1172/jci.insight.163360

**Published:** 2023-04-10

**Authors:** Henry S. Cheng, Rulin Zhuang, Daniel Pérez-Cremades, Jingshu Chen, Anurag Jamaiyar, Winona Wu, Grasiele Sausen, Aspasia Tzani, Jorge Plutzky, Jorge Henao-Mejia, Philip P. Goodney, Mark A. Creager, Marc S. Sabatine, Marc P. Bonaca, Mark W. Feinberg

**Affiliations:** 1 Department of Medicine, Cardiovascular Division, Brigham and Women’s Hospital, Harvard Medical School, Boston, Massachusetts, USA.; 2 Department of Cardiothoracic Surgery, Nanjing Drum Tower Hospital, The Affiliated Hospital of Nanjing University Medical School, Nanjing, China.; 3 Department of Physiology, University of Valencia, and INCLIVA Biomedical Research Institute, Valencia, Spain.; 4 Department of Pathology and Laboratory Medicine, and; 5 Institute for Immunology, Perelman School of Medicine, and; 6 Division of Protective Immunity, Department of Pathology and Laboratory Medicine, Children’s Hospital of Philadelphia, University of Pennsylvania, Philadelphia, Pennsylvania, USA.; 7 Heart and Vascular Center, Dartmouth-Hitchcock Medical Center and Geisel School of Medicine at Dartmouth, Lebanon, New Hampshire, USA.; 8 CPC Clinical Research, University of Colorado, Denver, Colorado, USA.

**Keywords:** Angiogenesis, Vascular Biology, Cardiovascular disease, Diabetes, Hematopoietic stem cells

## Abstract

Patients with peripheral artery disease (PAD) and diabetes have the highest risk of critical limb ischemia (CLI) and amputation, yet the underlying mechanisms remain incompletely understood. MicroRNA (miRNA) sequencing of plasma from diabetic patients with or without CLI was compared to diabetic mice with acute or subacute limb ischemia to identify conserved miRNAs. miRNA-KO mice on high-fat diet were generated to explore the impact on CLI. Comparison of dysregulated miRNAs from diabetic individuals with PAD and diabetic mice with limb ischemia revealed conserved *miR-181* family members. High-fat–fed, diabetic *Mir181a2b2*-KO mice had impaired revascularization in limbs due to abrogation of circulating Ly6C^hi^ monocytes, with reduced accumulation in ischemic skeletal muscles. M2-like KO macrophages under diabetic conditions failed to produce proangiogenic cytokines. Single-cell transcriptomics of the bone marrow niche revealed that the reduced monocytosis in diabetic KO mice was a result of impaired hematopoiesis, with increased CXCR4 signaling in bone marrow Lineage^–^Sca1^+^Kit^+^ (LSK) cells. Exogenous Ly6C^hi^ monocytes from nondiabetic KO mice rescued the impaired revascularization in ischemic limbs of diabetic KO mice. Increased *Cxcr4* expression was mediated by the miR-181 target, *Plac8*. Taken together, our results show that *MiR-181a/b* is a putative mediator of diabetic CLI and contributes to changes in hematopoiesis, monocytosis, and macrophage polarization.

## Graphical abstract

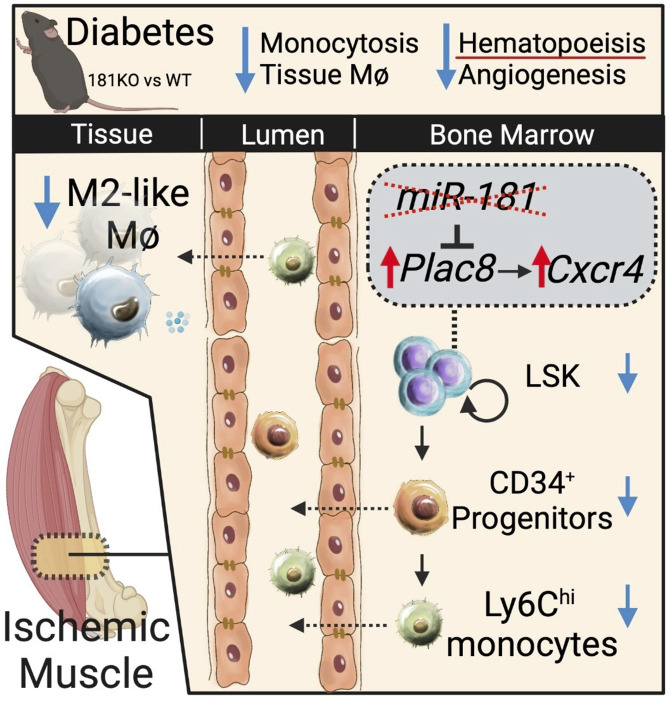

## Introduction

Peripheral artery disease (PAD) is the result of impaired blood flow to the lower extremities, affects more than 200 million people worldwide, and confers an increased risk of cardiovascular morbidity and mortality (1, 2). Though many patients are asymptomatic, others develop symptomatic manifestations of PAD such as intermittent claudication (discomfort and pain) with walking. A subset (1%–2%) of these patients with PAD will develop critical limb ischemia (CLI), the most severe manifestation of PAD (3). CLI is characterized by chronic resting pain, development of ulcers and gangrene, impaired wound healing, and its association with high risk of lower extremity amputation (10%–40%) and cardiovascular death (20%) within the first year after diagnosis (3–5).

Risk factors associated with progression to CLI include smoking, age, hypertension, dyslipidemia, chronic kidney disease, and diabetes mellitus (DM). In particular, patients with PAD and DM have a 20%–30% higher risk of cardiovascular and limb events than those without DM (6). Indeed, DM has been shown to impair angiogenesis, the growth of blood vessels from preexisting ones (7). Hyperglycemia can compromise several different cell types involved in angiogenesis, including endothelial cells (ECs) and leukocytes (8, 9). In murine experimental PAD, a plethora of studies have demonstrated a vital role for recruitment of monocytes and macrophages in promoting revascularization of ischemic tissues (10–13). However, the nodal regulators of this response remain poorly understood.

MicroRNAs (miRNAs) are small noncoding RNAs capable of mediating posttranscriptional repression of targeted mRNA transcripts. While a growing body of research is dedicated to understanding single miRNA-mRNA mechanisms in a given system, it is now becoming more appreciated that miRNAs modulate gene networks to convey biological changes (14, 15). These miRNA-biological pathways have been studied in different mechanistic aspects of experimental PAD, including inflammation, hypoxia, and angiogenesis (16). Given that diabetes is a major risk factor for development of CLI, focused studies exploring miRNAs in response to diabetic experimental PAD is of interest to inform new pathobiologically relevant signaling pathways and targets.

Herein, we establish a discovery platform to evaluate the pathophysiology of miRNAs dysregulated in patients with PAD and diabetes and murine experimental PAD with diabetes. From this we identified members of the *miR-181* family to be involved in macrophage polarization and recruitment in ischemic tissues to mediate hind limb reperfusion. Furthermore, we uncover what we believe is a novel relationship involving a *miR-181*/CXCR4 signaling axis and primitive hematopoietic stem cells (HSCs) under diabetic conditions that regulates monocytosis.

## Results

### Identification of miR-181 family dysregulation in experimental PAD in diabetic mice and in patients with PAD and diabetes.

**Figure 1:**
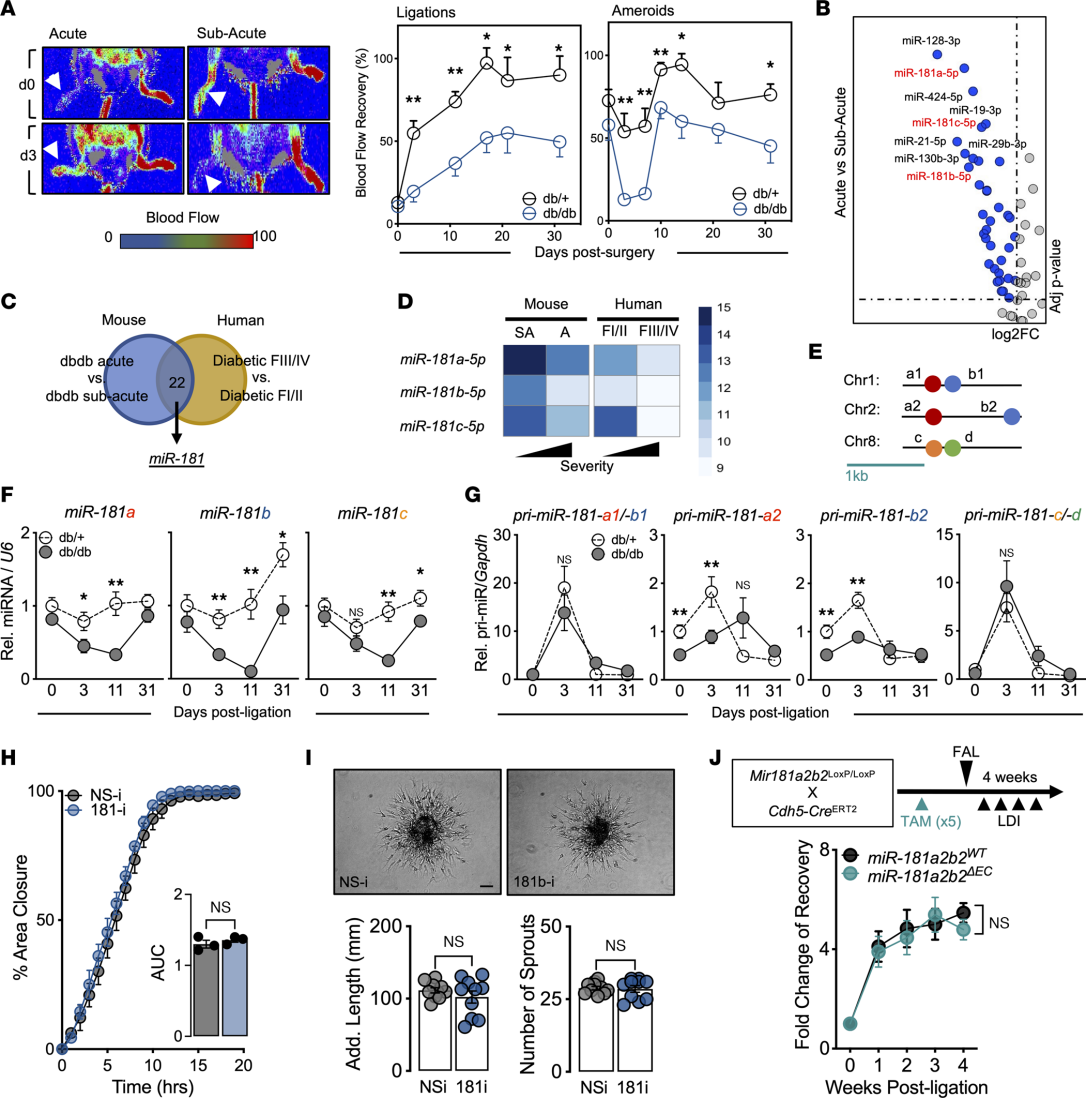
Identification of *miR-181* family dysregulation in experimental PAD in diabetic mice and in PAD individuals with diabetes mellitus. (**A**) Left: Representative images from laser Doppler imaging (LDI) of *db/+* and *db/db* mouse hind limbs 3 days after femoral artery ligation (FAL; acute ischemia) or ameroid constrictor implantation (subacute ischemia). Right: Quantification of blood flow recovery (surgical limb/contralateral limb). Significance was assessed within time points by unpaired, 2-tailed Student’s *t* test (*n* = 5–17). (**B**) Volcano plot highlighting downregulated miRNAs in plasma comparing acute versus subacute ischemia in *db/db* mice. (**C**) Venn diagram indicating number of miRNAs commonly dysregulated from human and mice miRNA-seq analysis. F, Fontaine. (**D**) miRNA-seq log_2_-normalized counts of *miR-181* members in human and mouse platforms (*n* = 6–7 human, *n* = 3 mice). A, acute; SA, subacute. (**E**) Genomic location of *miR-181* clusters in mice (*Mir181a1b1*, *Mir181a2b2*, and *Mir181cd*). (**F**) Mature *miR-181a/b/c* expression normalized to *U6* in ischemic gastrocnemius of *db/+* and *db/db* mice at different time points after FAL. Comparison between groups at specific time points by unpaired, 2-tailed Student’s *t* test (d0, *n* = 11–12; d3, *n* = 7–12; d11, *n* = 5–6; d31, *n* = 4–7). (**G**) Primary *miR-181-a1/b1*, *-a2*, *-b2*, and *-c/d* expression normalized to *Gapdh* in ischemic gastrocnemius of *db/+* and *db/db* mice at different time points after FAL (comparison statistics similar to **F**). (**H**) HUVECs transfected with 50 nM nonspecific (NS-i) or *miR-181* (181-i) inhibitors prior to scratch assay. Images captured every hour to determine area closure and area under curve (AUC) quantified and compared using unpaired, 2-tailed Student’s *t* test (*n* = 3). (**I**) Top: Representative images of 3D spheroids composed of HUVECs transfected with 50 nM NS-i or 181-i. Scale bar: 200 μm. Bottom: Additive sprout lengths and numbers quantified and compared using unpaired, 2-tailed Student’s *t* test (*n* = 10). (**J**) Top: Schema showing the generation of tamoxifen-induced (TAM-induced) *miR-181a2b2^ΔEC^* mice and experimental setup. Bottom: Blood flow (surgical limb/contralateral limb) quantified by LDI, normalized to measurement immediately after surgery. Two-way ANOVA (*n* = 6–7). *P < 0.05; **P < 0.01.

Two different types of surgeries were performed on the femoral artery to establish experimental PAD in mice with diabetes (*db/db*): (a) acute ischemia induced by femoral artery ligation (FAL) or (b) subacute ischemia induced by insertion of 2 ameroid constrictors around the femoral artery (17–19). Both ischemic procedures render lower blood flow recovery in *db/db* mice compared with nondiabetic mice (*db/+*) and can be tracked using laser Doppler imaging (LDI) to monitor longitudinal blood flow reperfusion kinetics (Figure 1A). Furthermore, *db/db* mice subjected to the FAL procedure are more prone to foot and toe necrosis compared with those subjected to ameroid constrictors (no necrosis), indicating a more severe hind limb ischemic response. We next performed miRNA sequencing (miRNA-seq) on plasma collected from *db/db* mice 3 days after surgery. When comparing mice subjected to acute ischemia to those with subacute ischemia with an adjusted *P* value of less than 0.05 and fold change (FC) of greater than 1.5, 36 miRNAs were identified that met these criteria. Surprisingly, all 36 miRNAs were downregulated, suggesting global repression of small RNAs due to the severity of ischemia (Figure 1B).

Individuals with PAD and diabetes classified as Fontaine class III and IV (FIII/IV; typically defined as ischemic rest pain with or without ulceration or gangrene) are at higher risk of developing adverse limb events, including amputation, compared with Fontaine class I and II (FI/II; typically defined as asymptomatic or with intermittent claudication) (20). As such, we performed miRNA-seq on plasma from individuals with PAD and DM (Supplemental Table 1; supplemental material available online with this article; https://doi.org/10.1172/jci.insight.163360DS1). Analysis was performed comparing the high-risk (FIII/IV) group to the low risk (FI/II) group, indicating 397 dysregulated miRNAs (209 upregulated and 188 downregulated, adjusted *P* value < 0.05, FC > 1.5). Twenty-two miRNAs were dysregulated in both human and mouse data sets (Figure 1C). Among the 22 miRNAs, 13 have been characterized and interrogated in hind limb ischemia studies. We selected the *miR-181* family (*miR-181a-5p*, *miR-181b-5p*, and *miR-181c-5p*), as 3 of the 4 members were identified as downregulated in high-risk PAD groups of mice and humans (Figure 1D). Furthermore, to our knowledge the *miR-181* family has not been investigated in diabetic CLI, which presents a novel avenue to pursue.

The evolutionary conserved *miR-181* family, composed of 4 members, is transcribed from 6 alleles and is found on 3 different chromosomes (Figure 1E). To assess whether the plasma miRNAs captured reflect tissue expression, we performed quantitative PCR analysis of ischemic gastrocnemius tissue collected at different time points (3, 11, and 31 days) following FAL. Similar to plasma analysis, all 3 *miR-181* members were downregulated in *db/db* compared with *db/+* mice, with *miR-181b-5p* having the greatest difference (Figure 1F). The difference between *db/+* and *db/db* for mature *miR-181a* and *miR-181b* expression appeared to be reflected in the *miR-181a2* and -*b2* alleles, as significant differences were observed when measuring ischemic gastrocnemius tissues for the primary transcripts *pri-miR-181a2* and *pri-miR-181b2*, whereas there were no differences in *pri-miR-181a1/b1* or *pri-miR-181c/d* (Figure 1G).

Among a variety of different mouse and human cell lines, we found *miR-181b* expression to be enriched in ECs (Supplemental Figure 1A). Impairment of blood flow to ischemic tissues is in part dependent on dysregulation of endothelial function and angiogenesis. To determine the endogenous role of *miR-181b* in angiogenesis, we transfected human umbilical vein ECs (HUVECs) with locked nucleic acid (LNA) inhibitors for *miR-181b*. When compared with HUVECs transfected with nonspecific LNA inhibitors, we found no differences in scratch wound assays (Figure 1H) and 3D spheroid–sprouting assays (Figure 1I). Next, we evaluated the regulation of proliferation and apoptosis of HUVECs under hypoxic and high-glucose conditions when *miR-181b* is inhibited and found no differences (Supplemental Figure 1, B and C). In support of this, we cultured lung microvascular ECs from systemic *miR-181a2b2*–KO mice (~60% reduction in *miR-181a/b*; Supplemental Figure 1D) and evaluated proliferation and apoptosis under hypoxic and high-glucose conditions and also found no differences compared to control (Supplemental Figure 1, E and F). Lastly, we performed FAL on inducible EC-specific *Mir-181a2b2*–KO mice (~43% reduction in *miR-181b* in CD31^+^ cells; Supplemental Figure 1G) and found no difference in blood flow recovery (Figure 1J). Taken together, our results show that we have identified *miR-181* members (primarily *Mir-181a2b2*) to be downregulated in experimental PAD in diabetic mice and in high-risk patients with PAD and diabetes. However, endothelial *miR-181* is not likely involved in the recovery of blood flow following hind limb ischemic injury.

### Deficiency of miR-181a2b2 hinders blood flow recovery following hind limb ischemia in diabetic mice.

**Figure 2:**
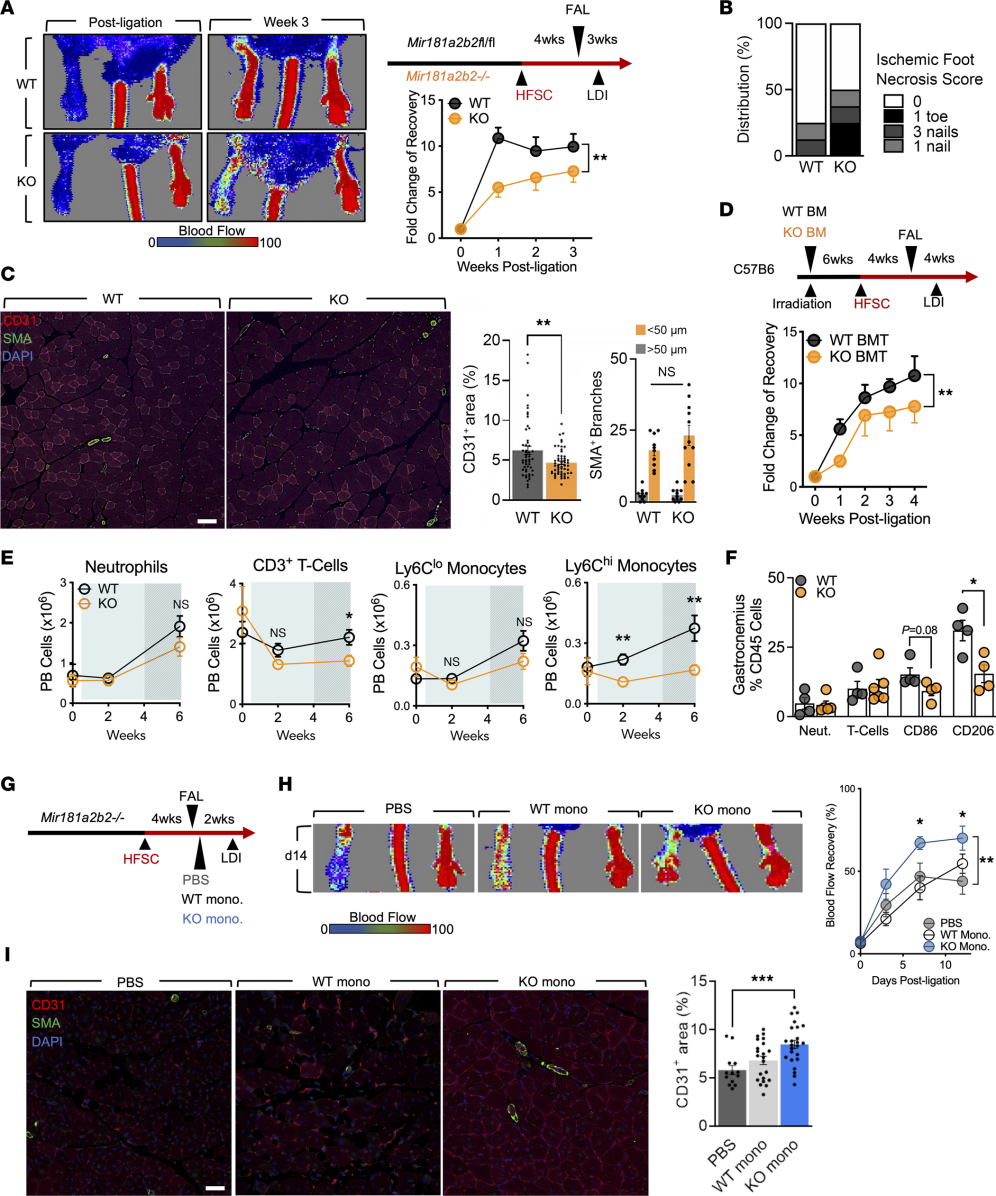
Diabetes hinders revascularization in *miR-181a2b2*–KO mice. (**A**) Left: Representative LDI images of hind limbs of WT and KO mice on HFSC diet immediately after FAL surgeries and 3 weeks later. Right: Schema showing experimental setup and quantification of blood flow (surgical limb/contralateral limb) quantified by LDI, normalized to measurement immediate after surgery. Two-way ANOVA (*n* = 10–11). (**B**) Necrosis score of ischemic foot 3 weeks after FAL. (**C**) Left: Representative immunofluorescence images of ischemic gastrocnemius stained for SMA (green), CD31 (red), and with DAPI (blue). Scale bar: 100 μm. Right: Quantification of CD31^+^ areas per field of view (5 images per sample, *n* = 10–11), and quantification of SMA^+^ arterioles with different diameters (*n* = 10–11). Unpaired, 2-tailed Student’s *t* test. (**D**) Top: Scheme showing generation of bone marrow–transplanted (BMT) mice and experimental setup. Bottom: Blood flow (surgical limb/contralateral limb) quantified by LDI, normalized to measurement immediately after surgery. Two-way ANOVA (*n* = 7–8). (**E** and **F**) Flow cytometric analysis of mice 2 weeks after FAL. Unpaired, 2-tailed Student’s *t* test. (**E**) Time course (0 week [chow-fed diet, *n* =3], 2 weeks [HFSC diet, *n* = 9], and 6 weeks [HFSC diet, *n* = 14]) of total circulating neutrophils, CD3^+^ T cells, Ly6C^lo^ and Ly6C^hi^ monocytes. (**F**) Ischemic gastrocnemius neutrophils (neut.), CD3^+^ T cells, CD86^+^ and CD206^+^ macrophages. Unpaired, 2-tailed Student’s *t* test (*n* = 4–5). (**G**) Schema of experimental setup with tail-vein delivery of PBS, WT monocytes, or KO monocytes given 1 day after FAL surgeries. (**H**) Left: Representative LDI images of KO mouse hind limbs 2 weeks after FAL surgeries. Right: Quantification of blood flow percentage (surgical limb/contralateral limb) quantified by LDI. Two-way ANOVA (*n* = 6–7). (**I**) Left: Representative immunofluorescence images of ischemic gastrocnemius stained for SMA (green), CD31 (red), and with DAPI (blue). Scale bar: 100 μm. Right: Quantification of CD31^+^ areas per field of view. Unpaired, 2-tailed Student’s *t* test (4 images per sample, *n* = 6–7). *P < 0.05; **P < 0.01; ***P < 0.001.

We next determined the role of *miR-181* in experimental PAD with diabetes. Placing systemic *miR-181a2b2*–KO and WT mice on a high-fat sucrose-containing (HFSC) diet (21) for 4 weeks before performing FAL reaffirmed our hypothesis that mice deficient in *miR-181* would suffer impaired hind limb perfusion (Figure 2A). Mice on the HFSC diet were tested for glucose and insulin tolerance and showed no significant differences between WT and KO (Supplemental Figure 2, A and B), suggesting the revascularization phenotype is independent of glucose metabolism. We also observed higher necrosis scoring in the ischemic feet of diabetic KO mice compared with diabetic WT mice (Figure 2B). Immunostaining of ischemic gastrocnemius demonstrated significantly less CD31^+^ area in KO mice (Figure 2C), which is in agreement with the LDI perfusion results. Quantification of SMA^+^ arterioles indicated no differences between WT and KO, which suggests impairment of revascularization is likely due to angiogenesis and not arteriogenesis (Figure 2C). Next, we performed bone marrow transplantation (BMT) of WT BM or KO BM into C57BL/6 mice followed by HFSC diet for 4 weeks and then FAL. Similarly, we observed impaired reperfusion of the hind limb in mice given KO BMT (Figure 2D), suggesting that in HFSC diet–fed mice the reperfusion phenotype is driven by leukocyte *miR-181a2b2*. To understand the transcriptional landscape underling these differences, RNA-seq and gene set enrichment analyses were performed on RNA derived from ischemic gastrocnemius of HFSC diet–fed KO and WT mice (Supplemental Figure 2, C and D). These ischemic gastrocnemius muscles manifested less leukocyte functionality and more diabetes and aging-related pathways (Insulin Receptor Signaling, Cardiac Hypertrophy, Sirtuin Signaling, and White Adipose Tissue Browning). However, insulin receptor signaling is influenced by inflammatory cytokines (22). Interestingly, many downregulated genes in the KO group suggested potential impact on leukocyte functionality, such as *Egr1*, *Pik3ap1*, *Itga4*, and *Srebf1* (Supplemental Figure 2D). Hence, we analyzed changes in circulating leukocytes on chow diet, 2 weeks on HFSC diet, and 2 weeks after FAL (total 6 weeks of HFSC diet). We observed a significant decrease in Ly6C^hi^ monocytes and CD3^+^ T cells in KO mice after 6 weeks of HFSC diet and 2 weeks of hind limb ischemia (Figure 2E). Furthermore, Ly6C^hi^ monocytes were modestly reduced after just 2 weeks of HFSC diet, suggesting that there may be diet-induced effects independent of ischemia. In gastrocnemius muscle of KO mice, there was also reduced accumulation of CD206^+^ macrophages and a nonsignificant trend for reduced CD86^+^ macrophages (Figure 2F). This decrease in macrophages was not due to changes in proliferation (Supplemental Figure 2E). In addition, we measured plasma levels of cytokines and discovered abrogated proinflammatory mediators in KO mice, such as CCL4, CXCL10, and CCL11 (Supplemental Figure 2F). While we are uncertain of the source of these cytokines in circulation and they are not predicted to be targets of *miR-181*, it suggests that *miR-181* may affect cytokine production indirectly or particular cytokine producing cells. Taken together, these results show that HFSC diet hinders revascularization in *miR-181a2b2*–KO mice after hind limb ischemia, with evidence suggesting altered monocyte functionality and inflammatory response.

Given the impaired proangiogenic role of *miR-181a/b* in experimental PAD and diabetes, we designed a rescue experiment to improve reperfusion in KO mice following HFSC diet and hind limb ischemia. The strategy aimed to increase the number of circulating Ly6C^hi^ monocytes that were found to be decreased in KO mice on HFSC diet and hind limb ischemia (Figure 2G). We supplemented HFSC diet–fed KO mice with FACS-isolated BM Ly6C^hi^ monocytes from either chow-fed WT or KO mice or PBS by tail vein injection 1 day following FAL (Figure 2G). Compared with the PBS-treated group, there was a remarkable improvement in reperfusion within 7 days after chow-fed KO Ly6C^hi^ monocyte transplantation (Figure 2H). Interestingly, WT Ly6C^hi^ monocytes did not significantly improve reperfusion compared to the PBS-treated group. Complementary to the LDI after reperfusion, cross-section immunofluorescent staining of CD31^+^ areas also indicated that KO Ly6C^hi^ monocyte treatment provided increased revascularization (Figure 2I). Taken together, our results provide a cell-based mechanism demonstrating that impaired hind limb perfusion in diabetic KO mice is rooted in the depletion of Ly6C^hi^ monocytes and their progenitor cells.

### Absence of miR-181a/b and diabetes alter BMDM functions.

**Figure 3:**
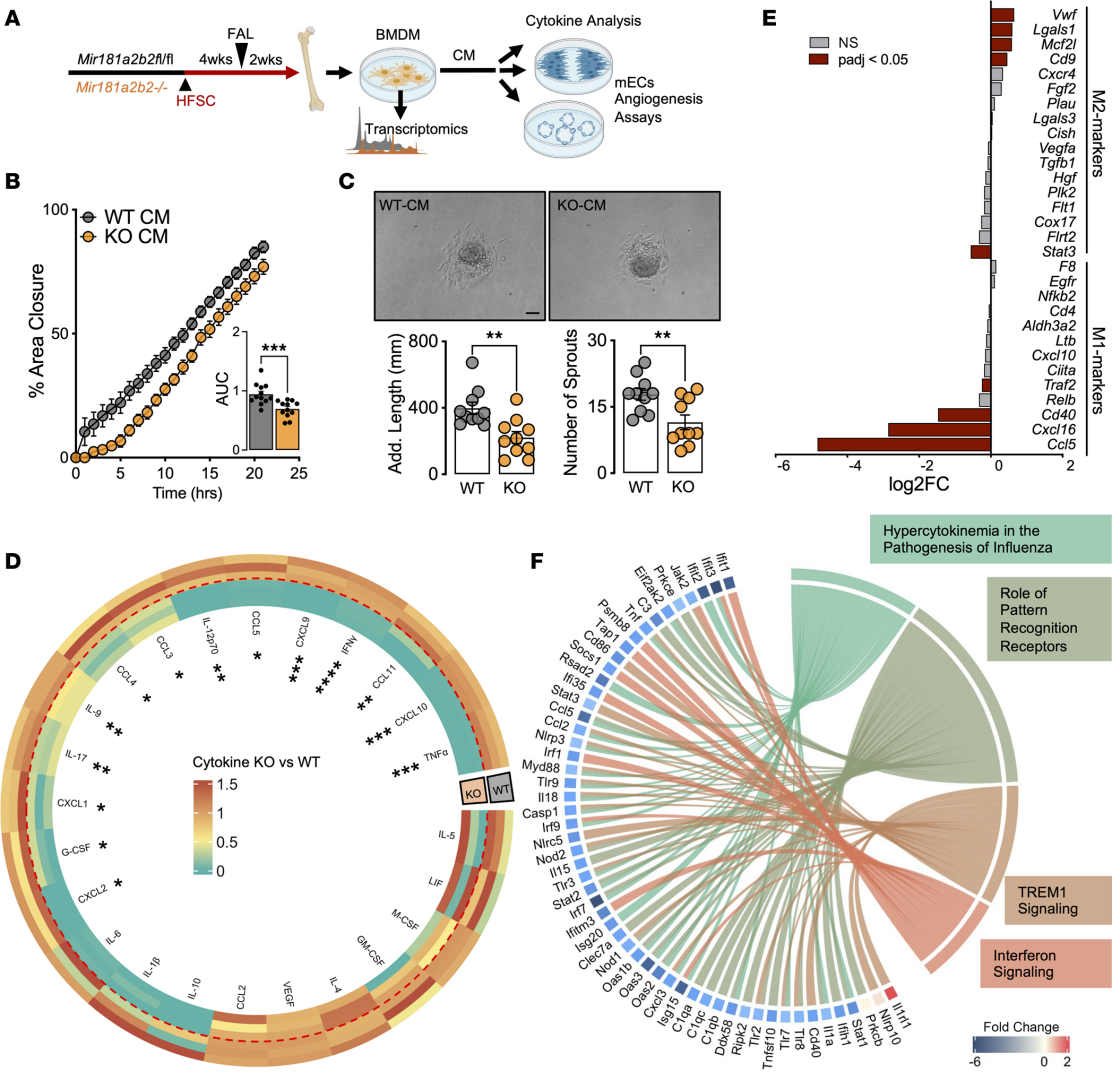
Diabetes impairs function of *miR-181a/b*–deficient macrophages. (**A**) Schema of preparation of BMDMs and experimental setup on HFSC diet. (**B**) Scratch assay performed using mECs treated with WT-CM or KO-CM. Significance of differences in AUC assessed using 2-tailed Student’s *t* test (*n* = 12). (**C**) Top: Representative images of 3D mEC spheroids treated with CM from WT or KO BMDMs. Scale bar: 200 μm. Bottom: Additive sprout lengths and numbers quantified and compared using unpaired, 2-tailed Student’s *t* test (*n* = 10). (**D**) Circular heatmap comparing M2-like macrophage cytokine and chemokine production in CM (*n* = 3). (**E**) List of proinflammatory (M1) and antiinflammatory (M2) expression from M2-like macrophages comparing KO versus WT. Results are from RNA-seq (*n* = 3). (**F**) Chord plot highlighting top dysregulated genes contributing to dysregulated signaling pathways. *P < 0.05, **P < 0.01, ***P < 0.001, ****P < 0.0001.

In order to investigate the downstream effects of monocytes, we next derived macrophages from the BM of WT and KO mice that were also subjected to 6 weeks of HFSC diet and FAL (Figure 3A). Conditional media (CM) collected from macrophages were transferred to murine ECs (mECs) and assessed for angiogenic response. CM from KO mice after HFSC diet and FAL impaired the angiogenic potential of mECs (Figure 3, B and C). When challenging these macrophages with IL-4 to induce an M2-like phenotype, KO BMDMs exhibited a loss of proangiogenic cytokine and chemokine production (Figure 3D and Supplemental Figure 3A). Conversely, when challenged with IFN-γ and lipopolysaccharide that induce an M1-like phenotype, no significant change in cytokine production occurred, with the exception of IL-4 (Supplemental Figure 3B). Transcriptome-wide analysis of the 3 varieties of macrophages (M0, M1-like, and M2-like) revealed dynamic changes only in M2-like macrophages between WT and KO under HFSC diet conditions (Supplemental Figure 3C). Interestingly, comparing classic macrophage polarization genes in M2-like macrophages indicated that KO BMDMs under HFSC diet were more skewed toward an M2-like phenotype compared with WT BMDMs (Figure 3E). The transcriptional changes in M2-like macrophages highlighted several interesting pathways downregulated when comparing KO to WT, including Hypercytokinemia in the Pathogenesis of Influenza, Role of Pattern Recognition Receptors, and TREM1 Signaling (Supplemental Figure 3D). The genes contributing to these pathways indicate a plethora of proangiogenic cytokines being repressed in KO macrophages (Figure 3F), in accordance with the supernatant cytokine analysis (Figure 3D). Furthermore, several prominent transcription factors involved in cytokine production were also shown to be repressed, such as *Jak2*, *Stat1*, *Stat2*, and *Stat3* (Figure 3F). Interestingly, the transcriptome-wide analysis also revealed a set of cell cycle–related genes upregulated in KO M2-like macrophages (Supplemental Figure 3E). Taking into consideration the marked depletion of macrophages in ischemic gastrocnemius in KO mice after HFSC diet and FAL (Figure 2, E and F), we measured BrdU incorporation to determine their proliferative capacity; however, there was no difference between WT and KO macrophages (Supplemental Figure 2E). Collectively, analysis of BMDMs from diabetic mice revealed that KO M2-like macrophages exhibit impaired cytokine and chemokine production, which partly explains their antiangiogenic effects on ECs.

### Deficiency of miR-181a/b impairs the abundance of hematopoietic stem and progenitor cells in diabetic mice.

**Figure 4:**
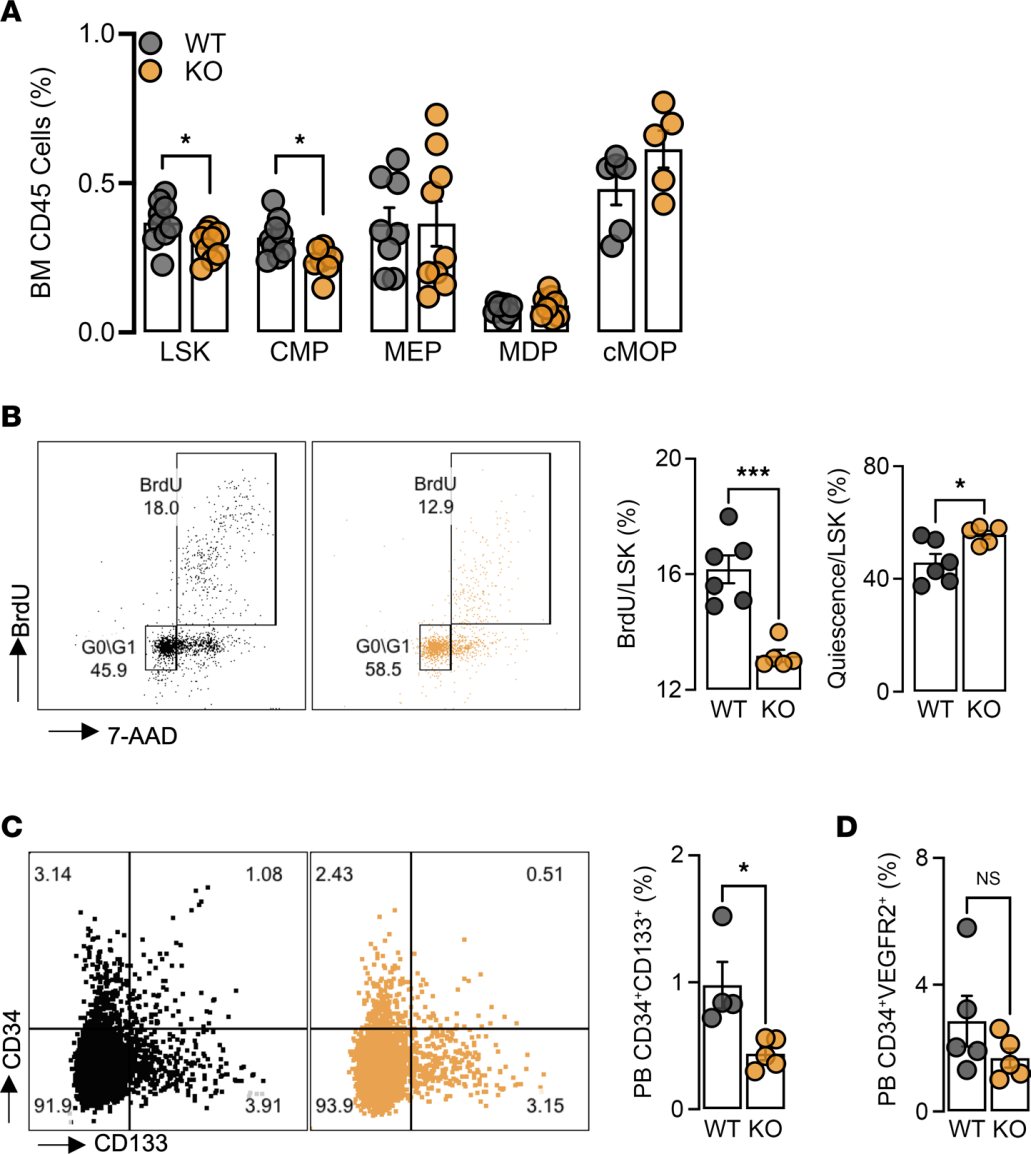
Diabetes in *miR-181a2b2*–KO mice impairs bone marrow hematopoiesis. Flow cytometric analysis of BM from femurs and peripheral blood 2 weeks after FAL and mice on HFSC diet for 6 weeks. (**A**) Hematopoietic stem and progenitor cells (HSPCs): Lineage^–^Sca1^+^c-Kit^+^ (LSK) cells, common myeloid progenitor (CMP), megakaryocyte-erythroid progenitor (MEP), monocyte–dendritic cell progenitor (MDP), and common monocyte progenitor (cMOP) (*n* = 5–8). (**B**) Left: Representative flow cytometric plots of BrdU and 7-AAD gated on WT (black) and KO (orange) LSK cells. Right: Quantification of proliferation (BrdU^+^) and quiescence (G_0_/G_1_ phase) of LSK cells (*n* = 5–6). (**C**) Left: Representative flow cytometric plots of CD34^+^CD133^+^ cells. Right: Quantification of CPCs in peripheral blood (PB) after 8 weeks on HFSC diet (*n* = 4–5). (**D**) Flow cytometric analysis of peripheral blood CD34^+^VEGFR2^+^ cells (*n* = 5). All statistical analyses performed with unpaired, 2-tailed Student’s *t* test. *P < 0.05; ***P < 0.001.

To explore whether stem cell progenitors contribute to the loss of monocytes with HFSC diet and hind limb ischemia, we examined hematopoiesis in the BM. KO mice showed a loss of the pluripotent Lineage^–^Sca1^+^c-Kit^+^ (LSK) cells and common myeloid progenitors (CMPs, CD34^+^CD16/32^+^CD115^+^), while other progenitors (megakaryocyte-erythroid progenitors [MEPs], monocyte–dendritic cell progenitors [MDPs], and common monocyte progenitors [cMOPs]) were unchanged compared to WT mice (Figure 4A). Furthermore, after pulsing with BrdU 2 hours prior to sacrifice, we found KO LSK cells to be less proliferative and more quiescent in G_0_ phase (Figure 4B). With the reduction in LSK cells and CMPs in the BM, we postulated that this may in turn affect circulating progenitor cells (CPCs), which are BM-derived circulating CD34^+^ cells that contribute to neovascularization of ischemic tissues through paracrine mechanisms (23). As such, we measured CPCs (CD34^+^CD133^+^) in HFSC diet–fed mice and found that KOs had approximately 50% less CPCs in peripheral blood compared with WT mice (Figure 4C). We also note that these cells are different from other angiogenic CPCs, such as EC progenitors, which are also CD34^+^ but express VEGFR2. Examination of CD34^+^VEGFR2^+^ progenitors showed no differences in circulating levels in the peripheral blood between WT and KO mice (Figure 4D). These findings suggest that *miR-181* regulates the maintenance of these pluripotent progenitors under diabetic conditions.

### CXCR4 signaling regulates proliferation of pluripotent progenitors.

**Figure 5:**
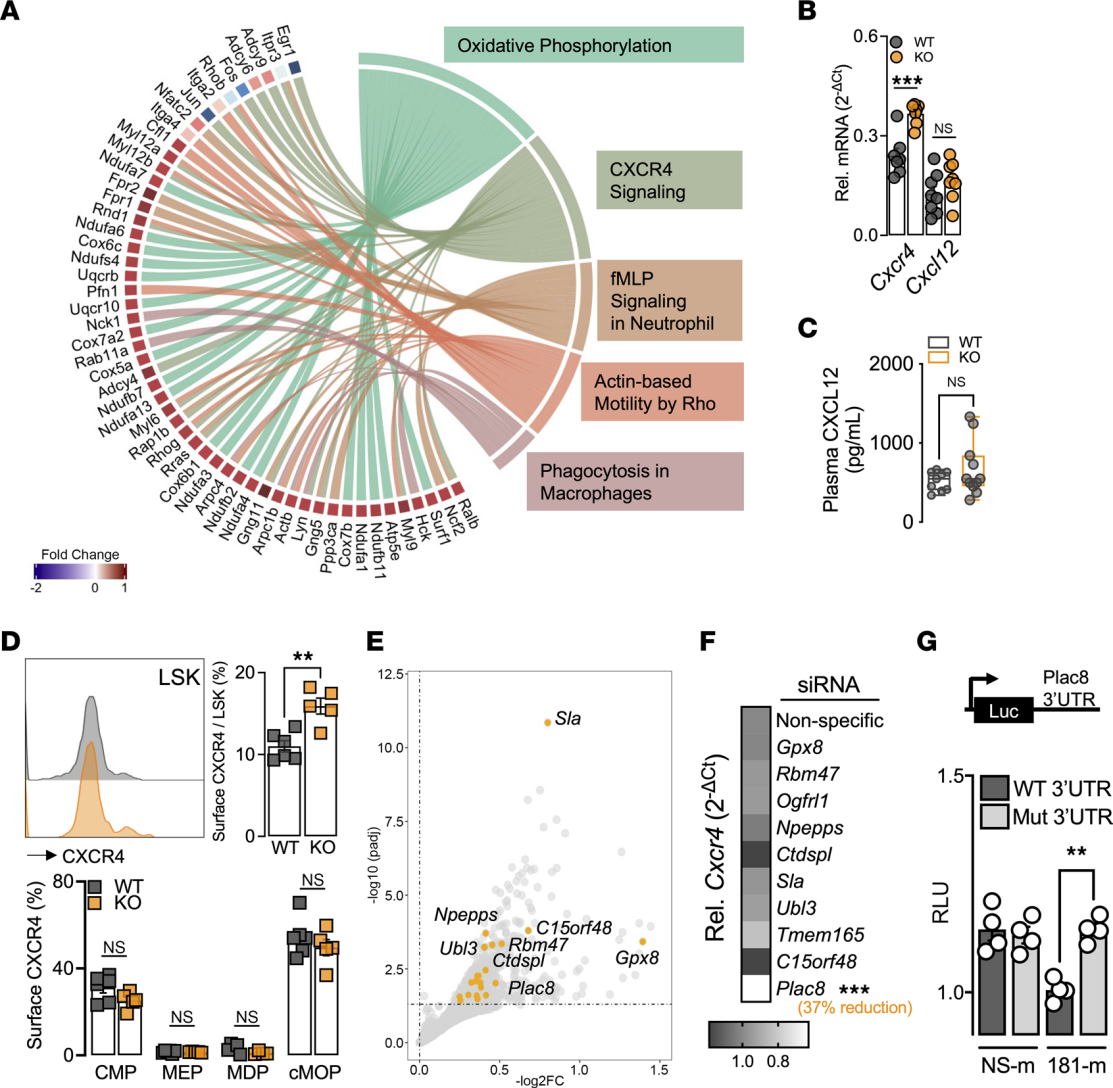
CXCR4 signaling regulates proliferation of pluripotent progenitors. (**A**) Chord plot highlighting top dysregulated genes and corresponding signaling pathways. (**B**) Gene expression of total BM for *Cxcr4* and *Cxcl12* normalized to *Hprt* (*n* = 7–8). (**C**) Plasma levels of CXCL12 measured by cytokine array (*n* = 9–11). (**D**) Surface CXCR4 expression. Left: Representative histogram plot of WT (gray) and KO (orange) LSK cells. Right: Quantification of LSK cells. Bottom: Quantification of HSPCs (*n* = 5–6). (**E**) Volcano plot highlighting top miR-181 predicted targets. (**F**) Gene expression of *Cxcr4* normalized to *Hprt* in BMDMs transfected with different siRNAs (*n* = 4). (**G**) Relative luciferase units (RLU) of WT *Plac8* 3′UTR and mutated (Mut) *Plac8* 3′UTR (TGAATGT → ACCTTCC) luciferase reporter assay with nonspecific mimic (NS-m) or miR-181b mimic (181-m) (*n* = 4). All statistical analyses performed with unpaired, 2-tailed Student’s *t* test. *P < 0.05; **P < 0.01; ***P < 0.001.

We next performed RNA-seq and pathway analysis on BM cells after HFSC diet feeding and hind limb ischemia (Figure 5A and Supplemental Figure 4A). The top dysregulated pathways were as follows: Oxidative Phosphorylation, IL-8 Signaling, Actin-based Motility by Rho, fMLP Signaling in Neutrophils, Actin Nucleation by ARP-WASP Complex, and CXCR4 Signaling. We recognized CXCR4 signaling to be of interest, as CXCR4 and its ligand CXCL12 (SDF-1) are known to decrease LSK cell proliferation and maintenance (24–26). Further examination into the dysregulated genes that contribute to CXCR4 signaling highlighted several transcription factors that are decreased in KO BM cells, such as *Jun*, *Fos*, and *Egr1* (Supplemental Figure 4, B and C), all of which have been shown to regulate hematopoiesis (27–29).

To distinguish between ligand and receptor inputs, we first measured gene expression and found *Cxcr4* significantly upregulated in KO BM cells, whereas no change was observed in the ligand *Cxcl12* (Figure 5B). In addition, there were no changes in circulating CXCL12 levels (Figure 5C). The interaction between CXCR4 and CXCL12 is also known to lead to monocyte retention in the BM and therefore could in part explain the decrease in circulating Ly6C^hi^ monocytes in KO mice under HFSC diet and hind limb ischemia. Hence, we also evaluated the different levels of Ly6C-expressing monocytes in the BM and found no differences between WT and KO (Supplemental Figure 4D). To determine the cells affected by upregulation of CXCR4 signaling, we measured cell surface expression of CXCR4 by flow cytometric analysis on different hematopoietic stem and progenitor cells (HSPCs). Moreover, KO LSK cells were the only cell type to express higher cell surface CXCR4, further supporting the decreased LSK cell numbers (Figure 5D).

Given that *Cxcr4* is not predicted to be a target of *miR-181*, we then overlapped upregulated genes from our RNA-seq data set to predicted targets of *miR-181* with IPA software (Figure 5E). Using siRNAs specific for upregulated potential *miR-181b* targets, we found that 1 candidate gene, placenta-specific 8 (*Plac8*), promotes the expression of *Cxcr4* (Figure 5F). Next, we confirmed *Plac8* to be a bona fide target of *miR-181* by luciferase assays with the *Plac8* 3′UTR (Figure 5G). Taken together, our results show that KO mice under HFSC diet and hind limb ischemia have fewer pluripotent LSK cells in the BM, likely due to their increased cell surface expression of CXCR4 and enhanced BM retention and quiescence. This induction of *Cxcr4* is mediated by the increased *miR-181* target *Plac8* under diabetic conditions.

### Single-cell resolution of stem and progenitor cells reveals impaired myelopoiesis in diabetic miR-181a2b2–deficient mice.

**Figure 6:**
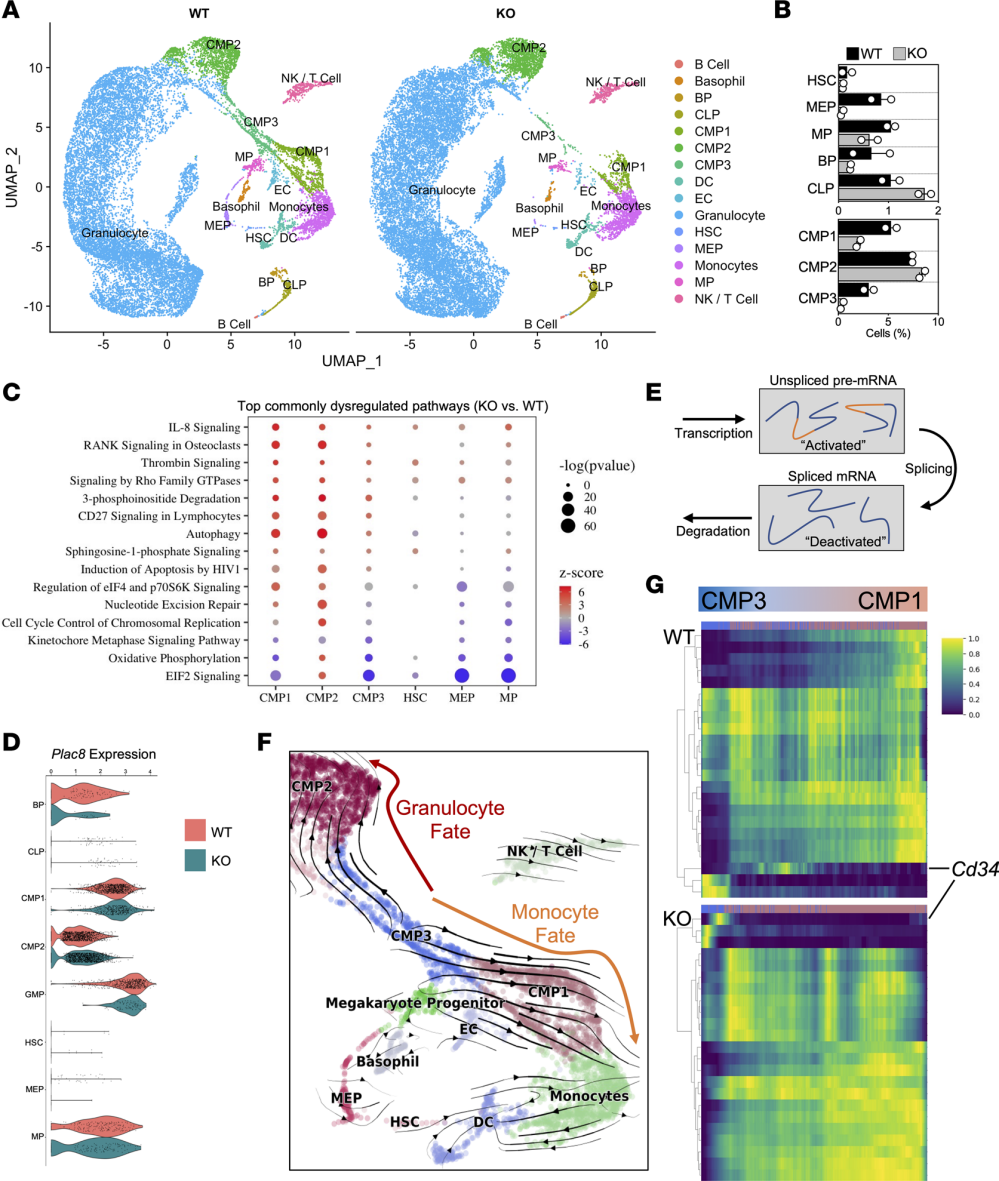
Single-cell resolution of stem and progenitor cells reveals impaired myelopoiesis in diabetic *miR-181a2b2*–deficient mice. (**A**) UMAP visualization of color-coded clustering of BM niche from 2 diabetic WT (*n* = 19,817 cells) and 2 diabetic KO (*n* = 22,072 cells) mice after FAL. Each dot is 1 cell. BM niche: B cell progenitor (BP), common lymphoid progenitor (CLP), common myeloid progenitor (CMP), dendritic cell (DC), endothelial cell (EC), hematopoietic stem cell (HSC), megakaryocyte-erythrocyte progenitor (MEP), megakaryocyte progenitor (MP), and natural killer (NK) cells. (**B**) Relative percentage of HSPCs per mouse BM (*n* = 2). (**C**) Top commonly dysregulated pathways across different progenitors organized by –log(*P* value). Positive and negative *z* score denotes whether the majority of genes that contribute to the assigned pathway is increased or decreased, respectively, compared with WT cells. (**D**) Violin expression plots of *Plac8* in different progenitor cells. (**E**) Modeling transcriptional dynamics captures transcriptional induction and repression (“activated” and “deactivated” phase) of unspliced pre-mRNAs, their conversion into mature, spliced mRNAs, and their eventual degradation. (**F**) Velocities derived from the dynamical model for hematopoiesis are visualized as streamlines in a UMAP-based embedding. (**G**) Top 20 gene expression dynamics along latent time from CMP3 toward CMP1 cells.

To further explore the impairment of hematopoiesis, we performed single-cell RNA-seq (scRNA-seq) on BM from HFSC diet–fed WT and KO mice subjected to FAL. Single-cell resolution revealed a striking discrepancy in several different cell clusters in KO BM (Figure 6A), especially for progenitor cell subsets, such as HSCs, MEPs, megakaryocyte progenitors (MPs), and CMP1 and CMP3 (Figure 6B). The majority of cells identified were granulocyte-like (i.e., neutrophils and eosinophils), which we grouped as “Granulocytes” (Supplemental Figure 7A). The HSPC population represented 19.5% of BM cells in WT mice, whereas they constituted 13.5% of KO BM cells (Supplemental Figure 7B). Differential expression and pathway analysis of the different progenitor clusters revealed that the top commonly dysregulated pathway was EIF2 signaling (Figure 6C). Interestingly, the progenitor subsets with lower cell numbers in KO BM had a negative *z* score for EIF2 signaling, while only the CMP2 cluster had a positive *z* score, suggesting that genes contributing to this pathway may be important for maintaining progenitor cell numbers (Supplemental Figure 7, C and D). Among the EIF2 signaling pathway were several ribosomal protein transcripts expressed markedly lower in BM KO CMP1, CMP3, and MP cells compared with their WT progenitor cell counterparts (Supplemental Figure 7D and Supplemental Table 6). Interestingly, we found only CMP2 cells had differentially expressed levels of *Plac8*, suggesting that the *miR-181*–*Plac8* interaction may occur in a cell-specific manner (Figure 6D).

RNA velocity is a new method that utilizes the abundance of unspliced (activated) and spliced (deactivated) mRNA transcripts derived from scRNA-seq to provide insights into the future state of individual cells, with applicability to cellular differentiation paradigms (30) (Figure 6E). Higher ratios of unspliced to spliced mRNA highlight the active transcription state within a given cell cluster. Interestingly, the BM progenitors that were decreased in KO mice had higher active transcription compared with the BM progenitors of WT mice (Supplemental Figure 7E). With this modeling, we could also determine that the progenitor trajectory of CMP3 to CMP1 leads to a monocyte fate, whereas CMP3 to CMP2 skews into a granulocyte fate (Figure 6F). Interestingly, CMP1 and CMP3 are progenitor cell populations depleted in the KO BM compared with WT BM. Mapping out the top dysregulated unspliced to spliced transcript ratios between CMP3 and CMP1 revealed many stem cell–related genes (i.e., *Cd34*; ref. 31) being disproportionately activated in KO CMP3s compared with WT progenitors where it is expressed during the transition from CMP3 to CMP1, suggesting dysregulation of CMP differentiation into monocytes (Figure 6G). Collectively, further investigation into the BM compartment at single-cell resolution revealed several different abnormalities in KO progenitors, such as decreased ribosomal protein gene expression, increased activated transcription, and dysregulated stem cell–related gene expression, most notably in the CMP3-to-CMP1 cells that may underlie the depletion of circulating Ly6C^hi^ monocytes observed in KO mice.

## Discussion

In this study, we explore the role of miRNAs in PAD and diabetes. Our approach using plasma from individuals with diabetes and PAD of increasing disease severity (Fontaine classification) allowed us to capture candidate miRNAs that might underlie the manifestation of CLI and diabetes. Leveraging the evolutionary conservation of miRNA biology, we mirrored our miRNA sequence screening with diabetic murine experimental PAD. Overlapping our human and mouse plasma sequence results captured a total of 22 miRNAs, including 13 that have been previously characterized in hind limb ischemia studies (*miR-15b-5p*, -*17-5p, -17-3p*, -*19a-3p*, -*19b-3p*, -*21-5p*, -*25-3p*, -*92a-3p*, -*93-5p*, -*106b-5p*, -*130a-3p*, -*146a-5p*, and -*223-3p*) (16), which confirmed that our approach enriched for revascularization-related miRNAs. We pursued *miR-181* as an intriguing candidate, given that 3 family members were all reduced in plasma and gastrocnemius muscle in ischemic models (Figure 1E). While *miR-181* members have been well characterized in several different cardiometabolic disorders, such as vascular inflammation (32, 33), atherosclerosis (34), glucose metabolism (35), and obesity (36, 37), its role in hind limb ischemia, PAD, and diabetes remained poorly understood.

Our study revealed an interesting role for the *miR-181* family in hind limb ischemia under diabetic conditions. The lack of blood flow recovery in diabetic KO mice is a result of deficient circulating Ly6C^hi^ monocytes, leading to a disparity in macrophage subgroups (Figure 2, G and H). This conclusion is supported in part by the improved hind limb perfusion seen after exogenous administration of Ly6C^hi^ monocytes from chow-fed KO mice to diabetic KO mice (Figure 2, G–I). Given that the KO phenotype stems from the BM and the myeloid population, we excluded the use of ectopic *miR-181* delivery, as targeted delivery to the BM is difficult and inconsistent. While there was minimal improvement in hind limb perfusion from WT monocytes from chow-fed mice, additional mechanisms may be involved in using the KO monocytes. Consistent with these findings, KO macrophages from diabetic mice impaired EC angiogenic functional properties (Figure 3, B and C). From RNA-seq and cytokine analyses, we also discovered that these macrophages are impaired when they become more M2-like with IL-4 induction, but not upon induction to an M1-like phenotype or in the unstimulated state (Figure 3, D and E). This may in part reflect a decrease in certain cytokines in circulation (Supplemental Figure 2F). This 1-sided impairment of macrophage polarization is seldom reported (38). Several mechanisms may explain these findings, including epigenetic reprogramming of macrophage progenitor cells through HFSC diet (39), changes in proximal mediators that impact differentiation, or alterations in key proteins that help sustain macrophage polarization (40–42). All of these and other hypotheses warrant further exploration, especially epigenetic regulation in macrophage polarization, as it may inform a wide range of disease states associated with diabetes and leukocytosis. While there was histological evidence of reduced neovascularization in the gastrocnemius of the diabetic KO mice, one limitation of the study is that we could not assess for arteriogenesis in adductor muscles due to technical considerations of performing femoral artery ligation that impacts this muscle group. Another potential limitation of this study is the use of BM transplantation instead of using a monocyte/macrophage-specific *miR-181a2b2*–KO mouse model. Future studies will be informative to further clarify the cell-specific leukocyte role for *miR-181a/b* in such a manner.

We also found *miR-181a/b* to be an important regulator of hematopoiesis in the setting of HFSC diet. Early studies using *Cxcr4*- or *Cxcl12*-deficient mice established the importance of HSPC maintenance of leukocytosis (43, 44). Under diabetic conditions in mice, decreased *Cxcl12* expression (not *Cxcr4*) determines the primitive hematopoietic maintenance in the BM niche and indirectly affects leukocytosis in the circulation (26). Here, we report a rare occurrence of dynamic change in *Cxcr4* only, specifically relegated to LSK cells. Increased cell surface expression of CXCR4 on LSK cells promoted quiescence, which aligns with other studies demonstrating that *Cxcr4^–/–^* LSK cells are more proliferative in the BM, resulting in decreased downstream myeloid and lymphoid cells (24, 45). We also observed decreased CMPs in KO BM, with no changes to proliferation (data not shown), an effect that likely results from upstream LSK cell depletion. Given that CMPs serve as a prominent monocyte progenitor cell lineage (46, 47), this cascade of depleted LSK cells and CMPs ultimately results in the reduction in circulating Ly6C^hi^ monocytes seen here. This is also supported at single-cell resolution, as we observed the monocyte-skewed progenitor lineage largely depleted in KO BM compared with WT BM (Figure 6). Interestingly, the CMP2 cluster displayed elevated *Plac8* expression in KO compared with WT without a change in *Cxcr4* expression, suggesting alternative mechanisms of PLAC8 independent of CXCR4. This may in part reflect the lack of depletion of this cell cluster compared with other progenitors. Furthermore, this also indicates that *miR-181* activity is not uniform among the different cell clusters within the BM. Other mechanisms revealed by scRNA-seq highlights hyperactive transcription (unspliced/spliced ratios) and impaired expression of ribosomal protein genes may be independently contributing to the depletion of progenitors. Hyperactive transcription has been associated with pluripotency in stem cells and global silencing as cells differentiate (48). The loss of particular ribosomal protein genes leads to ribosomopathies, such as Diamond-Blackfan anemia (49). Specifically, mutations in *RPS19* in patients with Diamond-Blackfan anemia affect proliferation and apoptosis of BM CD34^+^ progenitors (50, 51). Thus, the reduced expression of *Rps19* in CMP1, CMP3, HSC, and MP (but not CMP2 and MEP) may align with the reduced number of this CD34^+^ cell progenitor cell subset in our KO mice. While the scRNA-seq of the BM provided insights for specific progenitor subsets, we recognize that using whole BM rather than sorted progenitors limits our interrogation to LSK cells (i.e., HSCs). We also observed a decreased percentage of CPCs (CD34^+^CD133^+^CXCR4^+^), which are also reported to contribute to revascularization potential, adult vasculogenesis, and correlate with cardiovascular outcomes in DM patients (23, 52–54). Given their origins in the BM and expression of CD34 (similar to CMPs), this decrease in CPCs may also be a result of depleted LSK cells. While the importance of hind limb revascularization resulting from depleted circulating CPCs in KO mice is left unexplored in this study, the given observations are likely a reflection of the general loss of progenitor cells from the BM.

The increased *Cxcr4* expression is in part a result of alleviated repression of *Plac8* by *miR-181*. *Plac8* is known to regulate proliferation of different cancers (55, 56); however, it does not appear to affect HSCs and progenitor cells under nondiabetic conditions (57). Cai et al. performed their study in chow-fed mice and thus the *Plac8*-*Cxcr4* interaction may not be operative under nondiabetic conditions. Coexpression of *Plac8* and *Cxcr4* is found in circulating monocytes and monocyte-derived antigen-presenting cells in an experimental model of multiple sclerosis (58), suggesting chronic inflammation may be required for the *miR-181*/*Plac8*/*Cxcr4* axis to be activated. Furthermore, while our findings support the dynamic change in expression in *Cxcr4* from *Plac8*-knockdown experiments, the direct mechanisms underlying this PLAC8-CXCR4 interaction in specific cell types will require future investigation. Interestingly, PLAC8 has been shown to bind to the C/EBPβ promoter to induce expression in brown adipose tissue (59) and an isoform of C/EBPβ can promote the expression of *Cxcr4* (60), providing a possible connection between *Plac8* and *Cxcr4* expression. Further exploration of this interaction may provide insights into *Cxcr4* expression more broadly in health and disease.

**Figure 7:**
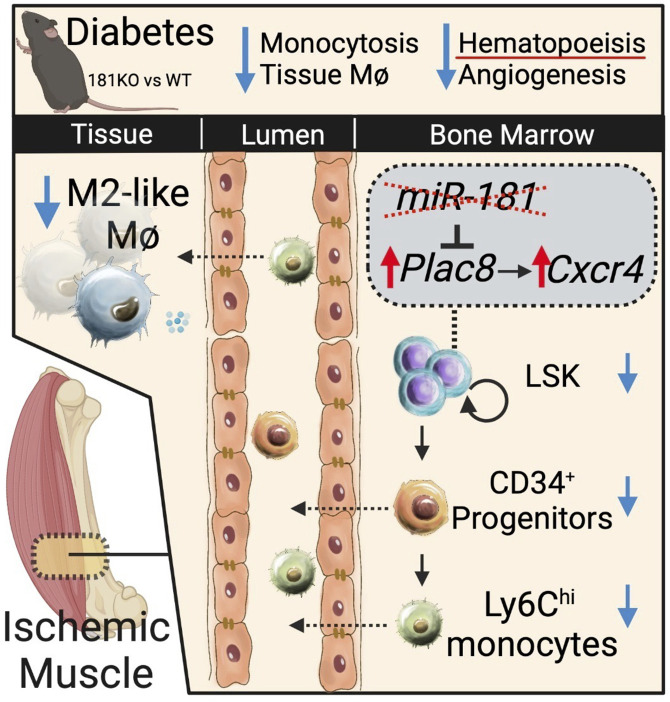
Schematic summary of hind limb ischemia in diabetic *miR-181a2b2*–KO mice.

In summary, our findings outlined an interesting cascade of events by *miR-181a/b* after hind limb ischemia–induced reperfusion (Figure 7). Under diabetic conditions, the absence of *miR-181a/b* alleviated *Plac8* and *Cxcr4* expression in primitive HSCs, resulting in reduced BM LSK cells, circulating Ly6C^hi^ monocytes, circulating CPCs, and tissue M2-like macrophages. This impaired leukocyte state in diabetes culminates in poor recovery from hind limb ischemia. However, this can be remedied with exogenous administration of Ly6C^hi^ monocytes from chow-fed mice. Furthermore, this study highlights the role of the *miR-181a/b*/CXCR4 signaling axis as a potential driver for progression of CLI under diabetic conditions.

## Methods

For detailed experimental methods, please see the Supplemental Methods section.

### Human samples.

Plasma for miRNA-seq was collected from the Thrombin Receptor Antagonist in Secondary Prevention of Atherothrombotic Ischemic Events (TRA 2°P)–TIMI 50 trial. All participants gave written consent for inclusion in the study. Characteristics of individuals are provided in Supplemental Table 1.

### Animal studies.

Studies were performed in *db/+* and *db/db* mice (The Jackson Laboratory), C57BL/6 mice (Charles River), inducible EC-specific *Mir181a2b2*-deficient mice, and systemic *Mir181a2b2*-deficient mice. All mice used were age-matched and sex-matched in all experiments and maintained under specific pathogen–free conditions at an American Association for the Accreditation of Laboratory Animal Care–accredited animal facility at the Brigham and Women’s Hospital. The animals were sacrificed at the end of each experimental time point. If an animal appeared to be sick or suffering, it was euthanized by CO_2_ asphyxiation. These methods are consistent with recommendations from the panel on Euthanasia of the American Veterinary Medical Association.

### Statistics.

Statistical analyses were performed using Prism version 7.0 (GraphPad Software Inc). Student’s *t* test was used to determine statistical significance between 2 groups. ANOVA with Bonferroni’s test was used to determine differences between more than 2 groups. Data are expressed as mean ± SEM, and results were considered as significantly different using *P* less than 0.05.

### Study approval.

All protocols concerning animal use were approved (no. 2016N000182) by the Institutional Animal Care and Use Committee at Brigham and Women’s Hospital and Harvard Medical School (Boston, Massachusetts, USA) and conducted in accordance with the NIH *Guide for the Care and Use of Laboratory Animals* (National Academies Press, 2011). All human patient sample collection conformed to the principles outlined in the Declaration of Helsinki and were approved by the institutional review board of Brigham and Women’s Hospital.

## Author contributions

MWF and HSC conceived the hypothesis. HSC, RZ, DPC, JC, AJ, WW, GS, and AT performed the experiments. HSC, RZ, DPC, JC, AJ, AT, JP, PPG, MAC, MSS, MPB, WW, and MWF designed experiments or interpreted the results. JHM provided critical reagents. HSC, DPC, and MWF wrote the manuscript.

## Supplementary Material

Supplemental data

## Version 1 02/23/2023

In-Press Preview

## Version 2 04/10/2023

Electronic publication
