# Supplementary material for: A miRNA/CXCR4 signaling axis impairs monopoiesis and angiogenesis in diabetic critical limb ischemia
Source: JCI Insight. 2023 Apr 10;8(7):e163360. doi: 10.1172/jci.insight.163360 (PMC10132154; doi:10.1172/jci.insight.163360)
Supplement: Supplemental data [file jciinsight-8-163360-s030.pdf]

SUPPLEMENTAL MATERIAL

A miRNA-CXCR4 signaling axis impairs monopoiesis and angiogenesis in diabetic critical limb ischemia

Running Title: *Cheng et al.; miR-181a/b regulates diabetic critical limb ischemia*

Henry S. Cheng, PhD[1#]; Rulin Zhuang, MD[1,2#]; Daniel Pérez-Cremades, PhD[1,3#]; Jingshu Chen, PhD[1#]; Anurag Jamaiyar, PhD[1]; Winona Wu, MD[1]; Grasiele Sausen[1], PhD, Aspasia Tzani, MD, PhD[1]; Jorge Plutzky, MD[1]; Jorge Henao-Mejia, MD, PhD[4,5,6]; Philip P. Goodney, MD, MS[7]; Mark A. Creager, MD[7]; Marc S. Sabatine, MD, MPH[1]; Marc P Bonaca, MD[8]; Mark W. Feinberg, MD[1*]

Methods

Human samples

Plasma for miRNA-sequencing was collected from the Thrombin Receptor Antagonist in Secondary Prevention of Atherothrombotic Ischemic Events (TRA 2°P)-TIMI 50 trial. All participants have given written consent to the inclusion in the study. Characteristics of human subjects are found in **Supplemental Table 1**. All human patient samples conform to the principles outlined in the Declaration of Helsinki.

Animal studies

Studies were performed in *db/+ db/db* mice (The Jackson Laboratory), C57Bl/6 mice (Charles River), inducible EC-specific *Mir181a2b2*-deficient mice, and systemic *Mir181a2b2*-deficient mice. All mice used were age-matched and sex-matched in all experiments and maintained under SPF conditions at an American Association for the Accreditation of Laboratory Animal Care-accredited animal facility at the Brigham and Women's Hospital. Animal protocol (#2016N000182) were approved by the Institutional Animal Care and Use Committee at Harvard Medical School and conducted in accordance with the National Institutes of Health Guide for Care and Use of Laboratory Animals.

Mir181a2b2$^{flox/flox}$ and *Mir181a2b2*$^{-/-}$ mice were used as previously described[64]. Mice expressing tamoxifen-inducible endothelial-specific VE-cadherin (VECad-CreER^{T2}; also known as Cdh5(PAC)-CreER^{T2}) was kindly provided by Ralf Adams[65]. Inducible EC-specific *Mir181a2b2*-deficient mice (*Mir181a2b2*$^{flox/flox}$, VECad-Cre-ER^{T2}) was generated by crossbreeding *Mir181a2b2*$^{flox/flox}$ mice and VECad-Cre-ER^{T2} mice. For induction of Cre activity, four weeks old male *Mir181a2b2*$^{flox/flox}$ mice carrying the VECad-Cre-ER^{T2} transgene were treated with either 4-hydroxytamoxifen (H6278, Sigma) (10 mg/kg) or the same volume of vehicle (corn oil) by intraperitoneal (i.p.) for 5 consecutive days to generate EC-specific *Mir181a2b2*-deficient mice (*Mir181a2b2*$^{\Delta EC}$) and flox-Cre control mice (*Mir181a2b2*^{WT}). For bone marrow transplantation models, recipient mice were subjected to whole-body irradiation (10

Gys) followed by injection of bone marrow donor cells (4 x 10^6 cells) by tail vein injection, followed by recovery for 6 weeks.

In some experiments, mice were placed on a high-fat sucrose-containing (HFSC) diet consisting of 58 kcal% fat and 28 kcal% carbohydrates for 4 weeks prior to surgery (D09071704, Research Diets). Insulin tolerance testing (ITT) and glucose tolerance testing (GTT) were performed after 4 weeks of HFSC diet feeding. Briefly, for GTT, mice were fasted for 12 h and then injected i.p. with D-glucose (G7201, Sigma, 1 g/kg). ITT was performed on mice after 6 h fasting and injected i.p. with recombinant human regular insulin (0.75 U/kg). Blood glucose levels were measured before injection and at 15, 30, 60, 90, and 120 min after glucose or insulin injection using glucometer.

The animals will be sacrificed at the end of each experimental point as described above. If an animal appears to be sick or suffering, it will be euthanized by CO2 asphyxiation. These methods are consistent with recommendations from the panel on Euthanasia of the American Veterinary Medical Association.

Hindlimb ischemia mouse models

Mice were subjected to two different surgeries to replicate critical limb ischemia: 1) femoral artery ligation, which causes immediate cessation of blood flow hence (Acute ischemia); and 2) ameroid constrictors which gradually expands from fluid adsorption inducing artery occlusion (Sub-acute ischemia). Briefly, mice were injected i.p. with 150 µl of 20% ketamine/5% xylazine in 0.9% saline. Once anesthetized, the right medial thigh to the suprapubic area was treated with a commercial emollient to remove fur and sterilized with Povidone iodine. Skin and fascia were dissected away to the femoral bed. In the acute hindlimb ischemia model, femoral artery and surrounding tissue was proximally and distally ligated with 7-0 Prolene sutures. The arterial bed in between sutures was cauterized. Abrogation of blood flow compared to the contralateral limb (<10%) was confirmed using a laser Doppler imager (Moor Instruments, UK). Sub-acute hindlimb ischemia model was performed using ameroid constrictors, which induce gradual femoral artery occlusion over 1-3 days. Two ameroid constrictors were placed on the femoral artery, one proximal to the lateral circumflex femoral artery and the second proximal to the bifurcation of the popliteal

and saphenous arteries. Both constrictors were positioned with the slot facing up, ensuring proper setting of the artery within the constrictor. Mice were sutured closed at the level of the fascia and subsequently, the skin. Sham-treated mice were treated the same way except once the femoral artery was visualized, the incision was closed without ligation of the femoral artery or ameroid constrictor addition. Percent blood flow recovery was calculated by comparing a ratio of ischemic paw to contralateral paw Doppler count profiles and normalized blood flow recovery was calculated by comparing the ratio of ischemic to contralateral paw Doppler count profiles to day 0 post-operative percent blood flow.

Endothelial Cell Isolation

Gastrocnemius muscles or lungs were grinded with scissors and digested by using 1 mg/ml Collagenase type 2 (Worthington Biochemical LS004177) and 1 mg/ml Dispase II (Roche, 04942078001) and incubated at 37°C for 40 minutes. Digestion was neutralized with DMEM/F12 medium containing 10% FBS, followed by centrifugation at 500g for 10 min at 4℃. The slurry was passed through cell strainers (Corning Falcon/Westnet). After centrifugation, the cell pellet was re-suspended in incubation buffer (PBS pH 7.2, 0.1% BSA, 2mM EDTA, 0.5% FBS). Endothelial cells were captured using magnetic Dynabeads (sheep antirat IgG, Invitrogen, 00412289) conjugated with rat anti-mouse CD31 antibody (BD Biosciences, 557355) at a ratio of 5:1 Dynabeads/antibody and allowed to tumble at 4°C for 20 minutes. The slurry of lysate and Dynabead/antibody mixture was bound on a Dynamag-2 Magnet (Invitrogen) for 1 minute and the supernatant was collected as a non-endothelial cell fraction. The beads containing bound endothelial cells were then washed on the Dynamag-2 Magnet five times using wash buffer (PBS pH 7.2, 0.1% BSA) and the resultant pellet was collected as an endothelial cell fraction.

Plasma miRNA sequencing

The EdgeSeq miRNA Whole Transcriptome Assay from HTG Molecular Diagnostics, Inc. (AZ, USA) was used to measure miRNA expression in plasma from human donors and mice. The HTG EdgeSeq system combines quantitative nuclease protection assay chemistry with a next-generation sequencing platform to enable the semi-

quantitative analysis of 2,083 human miRNA transcripts in a single assay. Fifteen microliters of plasma were used for extraction-free sample processing and quantitative nuclease protection assay using the EdgeSeq processor (HTG Molecular Diagnostics, Inc.). The libraries were sequenced using Illumina NextSeq, and data were parsed through HTG EdgeSeq before count data were assessed for quality and analyzed using R.

Bulk RNA-Seq analysis

RNA-Seq analysis was performed after ribodepletion and standard library construction using Illumina HiSeq2500 V4 2x150 PE (Genewiz). All samples were processed using an RNA-seq pipeline implemented in the bcbio-nextgen project (https://bcbionextgen.readthedocs.org/en/latest/). Raw reads were examined for quality issues using FastQC (http://www.bioinformatics.babraham.ac.uk/projects/fastqc/) to ensure library generation and sequencing were suitable for further analysis. Trimmed reads were aligned to UCSC build mm10 of the mouse genome and augmented with transcript information from Ensembl releases 86 (H. sapiens) using STAR. Alignments were checked for evenness of coverage, rRNA content, genomic alignment context and other quality checks using a combination of FastQC and Qualimap. Counts of reads aligning to known genes were generated by featureCounts. Differential expression at the gene level was called with DESeq2. Total gene hit counts and CPM values were calculated for each gene and downstream differential expression analysis between specified groups was performed using DESeq2 and an adapted DESeq2 algorithm that excludes overlapping reads. Genes with adjusted FDR< 0.05 and log2fold-change (>1.5) were called as differentially expressed genes for each comparison. The mean quality score of all samples was 35 with a range of 35,000,000-67,000,000 reads per sample. All samples had at least >93% of mapped fragments over total fragments.

Pathway enrichment analysis and miRNA target prediction

Differentially expressed genes (DEGs) were identified as being at least 2.0-fold change and adjusted p-value < 0.05. DEGs were subjected to gene set enrichment

analyses by using Ingenuity Pathway Analysis (IPA winter release Dec 2020, Qiagen) software. For canonical pathway analysis, IPA (fall release Dec 2020) was used. The pathway activity (*Z* score) was computed to determine whether the activity of canonical pathways is increased or decreased on the basis of differentially expressed genes in the data sets. The significant values for the canonical pathways were calculated by Fisher exact test. Visualization of pathway enrichment analysis were performed as dotplot using (ggplot2 package) and circos plot (circlize package[66]) in R program. IPA microRNA Target Filter tool was used for miRNA target prediction. Different miRNA target prediction programs (TargetScan, miRecords, Ingenuity Knowledge Base and TarBase) filtered our miRNA-mRNA pairings. Confidence filter was used by selecting both experimentally observed and predicted target correlations.

Cell Culture and Transfection

Human umbilical vein endothelial cells (HUVECs; Lonza) were cultured in endothelial cell growth medium EGM-2 (Lonza, CC-3162). Cells that were utilized for experiments were passaged no more than six times. bEnd.3 cells (ATCC, CRL-2299) were cultured in Dulbecco's Modified Eagle Medium/F12(1:1) (DMEM; Gibco, 11320-033) supplemented with 10% fetal bovine serum (FBS) and 1% Penicillin-streptomycin (P/S). HEK293T cells (ATCC, CRL-3216) were cultured in Dulbecco's Modified Eagle Medium (DMEM) supplemented with 10% FBS and 1% P/S. For culture bone marrow-derived macrophages (BMDMs), bone marrow was isolated from the femur mice and cultured in Iscove's Modified Dulbecco's Medium (IMDM; Sigma, I3390) supplemented with 20 ng/ml recombinant mouse M-CSF (macrophage colony stimulation factor) (R&D Systems, 416-ML-10), 10% FBS and 1% P/S. Medium was changed every 3 days and cells were used after 7 days in culture. Stimulation to M1-like macrophages with 100 ng/ml lipopolysaccharide and 50ng/ml interferon-gamma for 48 hours. Stimulation to M2-like macrophages with 20 ng/ml interleukin-4 for 48 hours. Transfection was performed using Lipofectamine 2000 (Invitrogen) as described in the manufacturer's protocol. Negative control inhibitor (AM17011), hsa-miR-181b-5p inhibitor (4464084, MH12442), negative control siRNA (AM4611) siPLAC8 (s106863), siGPX8 (s87986), siSLA (s73822), siC15orf48 (s119453), siRBM47 (s110729), siCTDSPL (s87642), siNPEPPS (s72247), siOGFRL1 (s88581), siUBL3 (s76917),

siTMEM165 (s75367) were all purchased from Ambion and used for transfection at 50 nM (HUVECs) or 100 nM (BMDMs).

RNA Isolation and real-time quantitative PCR

Total RNA was extracted by using Trizol reagent following the manufacturer's protocol (Invitrogen, 15596-026). The concentration and quality control of RNA was examined using NanoDrop 2000 (ThermoFisher). Isolated RNA was reverse transcribed using miScript reverse transcription kit from Qiagen (218061) according to the manufacturer's instructions. miScript SYBR Green PCR Kit (Qiagen, 218073) was used for quantitative real-time PCR analysis with the AriaMx real-time PCR system (Agilent Technologies) following the manufacturer's instructions. mRNAs expression levels were normalized to *Hprt* and were calculated using $2^{-\Delta Ct}$ method. miRNAs were normalized to *U6* (**table S2**). cDNA was produced using High-Capacity cDNA Reverse Transcription Kit (Thermofisher, 4368814) to detect primary-miR-181 transcripts (TaqMan, a1/b1 Mm03307120_pri; a2 Mm03306417_pri; b2 Mm03307414_pri; c/d Mm03308358_pri) and normalize to *Gapdh* (TaqMan, Mm99999915_g1). Subsequent RT-qPCR was performed using GoTaq qPCR Master Mix (Promega). List of primers in **table S3**.

Immunofluorescence staining

For immunofluorescence staining, cells were fixed in 4% PFA (Boston Bio Products) for 24h and embedded in paraffin for sectioning.

Slide sections were blocked with 5% donkey serum (Jackson ImmunoResearch Lab) for 1 h and then incubated with primary antibodies, CD31 (1:50, Dianova, DIA310) and αSMA (1:500, Sigma-Aldrich, A5228) overnight at 4 ºC. Slides were washed and incubated with conjugated secondary antibodies (Jackson ImmunoResearch Lab) Cy3 conjugated donkey anti-rat secondary antibody (1:300, Cat#: 712-165-153) and Alexa 647 conjugated donkey anti-rabbit secondary antibody (1:300, Cat#: 711-605-152) and Alexa 488 conjugated donkey anti-rabbit secondary antibody (1:300, Cat#: 711-545-152) for 90 min at room temperature. Cell nuclei were stained with 4′,6-diamidino-2-phenylindole (DAPI). Immunofluorescence imaging was performed by BIDMC

confocal imaging and IHC core facility. Images were acquired on a Carl Zeiss LSM 880 confocal microscope using Zen black software version 2.3 SP1 (BIDMC confocal imaging and IHC core facility). Objective lenses 10x 0.45 NA and 20x 0.8 NA were used for image acquisition.

In vitro functional assays

Scratch assays were performed seeding 20,000 HUVECs per well in 35 mm µ-dish glass plates (Ibidi, 501149017) in EGM2 media containing growth factors and 50 ng/µl VEGF (R&D Systems, 293-VE/CF). When cells were confluent, well-dividers were removed and time 0 image was obtained. Imaging was taken every hour up to 24 hours using CytoSMART Omni (Cytosmart). For EC spheroid sprouting assay, HUVECs were cultured overnight in hanging drops on nonadherent plastic dishes in EBM-2 medium with 0.2% methylcellulose (Sigma-Aldrich) using 1,000 cells/spheroid. B.End3 spheroids were performed similarly with DMEM media in place of EBM-2. Spheroids were embedded in a collagen matrix and incubated for 24 h. Number of sprouts and total sprout length of 10 spheroids per condition were used for data analysis by using NIH ImageJ software.

Proliferation assays were performed by culturing HUVECs or primary lung microvascular ECs in EGM-2 containing 25mM of mannitol or D-glucose for 48 hours and seeding 4,000 cells per well in 96-well plates. 24 hours later, cells were labeled with BrdU labeling reagent and placed in either in normoxic conditions (21% O2) or hypoxic conditions (2-3% O2) for 8 hours. Cells were subsequently fixed and quantitated using the Cell Proliferation ELISA BrdU Colorimetric Kit according to the manufacturer's instructions (Roche, 11647229001).

Apoptosis assays were performed by culturing HUVECs or primary lung microvascular ECs in EGM-2 containing 25mM of mannitol or D-glucose for 48 hours and seeding 20,000 cells per well in 96-well plates. Cells were placed in either in normoxic conditions (21% O2) or hypoxic conditions (2-3% O2) for 24 hours. Cells were subsequently assessed for viability and apoptosis using the ApoLive-Glo Multiplex Assay according to the manufacturer's instructions (Promega G6410).

Luciferase Reporter Assay

Plac8 3'UTR reporter generated from Genecopoeia (HmiT103805-MT06; NM_016619.2). HEK293T cells co-transfected with 1µg luciferase plasmid vector with 50 nM miR-181b mimic or non-specific mimic for 24 hours. Analysis of luciferase activity with Dual-Luciferase Reporter Assay System (Promega, E1910) and standard 96-well plate reader.

Cytokine analysis

Mouse plasma and supernatant from cultured BMDM were collected and subjected to ELISA using Mouse Cytokine Discovery Assay (Eve Technologies).

Mononuclear cell preparation and flow cytometry

Peripheral blood, bone marrow and gastrocnemius muscle were used for characterization of leukocyte cell populations by flow cytometry. Peripheral blood was drawn from the right ventricle to EDTA contained tube, and cells were filtering through 70µm strainers after lysis of erythrocytes by using RBC Lysis Buffer (eBioscience, 00-4333-57). For bone marrow, one femur was crushed with a mortar and pestle and homogenized by passing through a 40µm strainer. For gastrocnemius muscle, after being washed with pre-cold 1x PBS, tissue was placed in the digestion buffer (1 mg/ml collagenase type 2) (Worthington, LS004177), 1 mg/ml dispase II (Roche, 04942078001) and minced with scissors. Then, tissues in digestion solution were incubated at 37°C for 30 min at a speed of 200 rpm shaking. The solution was passed through 70µm strainers and then centrifuged at 500x*g* for 10 minutes at 4°C. After that, samples were resuspended to obtain single cell suspensions for next step.

After preparing the single cell suspension according to the above methods, the samples from murine PBMC, bone marrow, and gastrocnemius sequentially filtered through 40µm strainers. Followed the manufacturer's instructions, added appropriately fluorescently labeled antibodies at predetermined optimum concentrations and incubated on ice for 20 minutes in the dark for cell-surface staining. After washing with PBS, centrifuging at 350xg for 5 minutes, samples were resuspended for flow

cytometric analysis (BD FACS Analyzer LSR, or BD FACS Analyzer Symphony). For detecting the cell proliferation in vivo, BrdU Flow Kits (BD Pharmingen, 559619) was used. Two hours before harvesting, 10 mg/mL solution of BrdU in sterile 1X DPBS was intraperitoneal injected. After staining with fluorescent antibodies specific for cell surface markers, following the manufacturer's instructions, fix and permeabilize the cells, and then stain with BrdU and 7-AAD. After washing, samples were resuspended for flow cytometric analysis. Gating strategy found in **figure S5**.

The antibodies for flow cytometry were attached in **table S4.** All the flow data analysis were analyzed by FlowJo 10.7.1.

Ly6C^{hi} monocyte purification and infusion

After collecting bone marrow by crushing the femur with a mortar and passing through 40μm strainers, monocytes from miR-181 KO and WT mice were enriched by using monocyte isolation kit (BM) (miltenyibiotec, 130-100-629). Purified monocytes were stained with anti-CD45, anti-CD115 and anti-Ly6C antibodies for sorting. Live CD45$^+$CD115$^+$Ly6C^{hi} cells were sorted by FACS Sorter Aria II.

One day post-ligation, 3x10^5 sorted Ly6C^{hi} monocytes from WT and KO mice were injected by tail vein to HFSC diet-fed KO mice. After 3, 7 and 14 days, blood flow was detected by using a laser Doppler imager and the tissue samples were collected to analyze further after 14 days.

Single-Cell RNA Sequencing

All portions of the scRNAseq workflow (single-cell suspension preparation, library preparation, quality control PCR) were performed at the biopolymer facility in Harvard Medical School and sequencing was performed by Novogene, Inc. Bone marrow cells suspension samples were diluted to target 10,000 cells for capture. The cells were processed using a 10X Genomics microfluidics chip to generate barcoded Gel Bead-In Emulsions according to manufacturer protocols. Indexed single-cell libraries were then created according to 10X Genomics specifications (Chromium Next GEM Single Cell 3' v3.1-Dual Index Libraries). Samples were multiplexed and sequenced in pairs on an Illumina HiSeq 4000 device.

Single-Cell RNA Sequencing Data Analysis

The sequenced data were processed into expression matrices with the Cell Ranger Single-cell software 6.1 (https://support.10xgenomics.com/single-cell-gene-expression/software/pipelines/latest/using/count) on the Harvard Medical School O2 cluster. Raw base-call files from HiSeq4000 sequencer were demultiplexed to first generate FASTQ files using the cellranger mkfastq pipeline. Subsequently, the reads were aligned to the mouse transcriptome (mm 10–3.0.0), cell barcodes and unique molecular identifiers (UMI) were filtered and corrected using the cellranger count pipeline. The final output filtered expression matrices were imported into the Seurat package in R and built into Seurat objects using the CreateSeuratObject function. DoubletFinder was used for removing potential doublets in the single-cell data. Filtering during this step included only genes detected in >3 cells, cells with >500 distinct genes and >500 UMI. Cells with >10% mitochondrial percentage were excluded. Cell types was identified based on gene markers for each type of cells (Table 2). Data normalization, scaling, and regression by mitochondrial content were then performed using the SCTransform command under default settings in Seurat. Principal component analysis and nonlinear dimensional reduction using Uniform Manifold Approximation and Projection (UMAP) was performed. Cell clustering was then assessed across a range of predetermined resolution scales to ensure separation of known major BM cell types without excessively sub-clustering. The enriched cell marker for each cluster of BM cell were shown in UMAP plots (fig. S6, table S5). The FindAllMarkers function in Seurat was applied to performs parallel differential expression testing of all cells within a cluster versus all other cells in the data set via nonparametric Wilcoxon rank-sum test using default parameters.

Data availability

All relevant data are available from the authors. The RNA-seq data are accessible at: https://www.ncbi.nlm.nih.gov/geo/query/acc.cgi?acc=GSE196566. Source data are provided with this paper.

Supplemental Figures

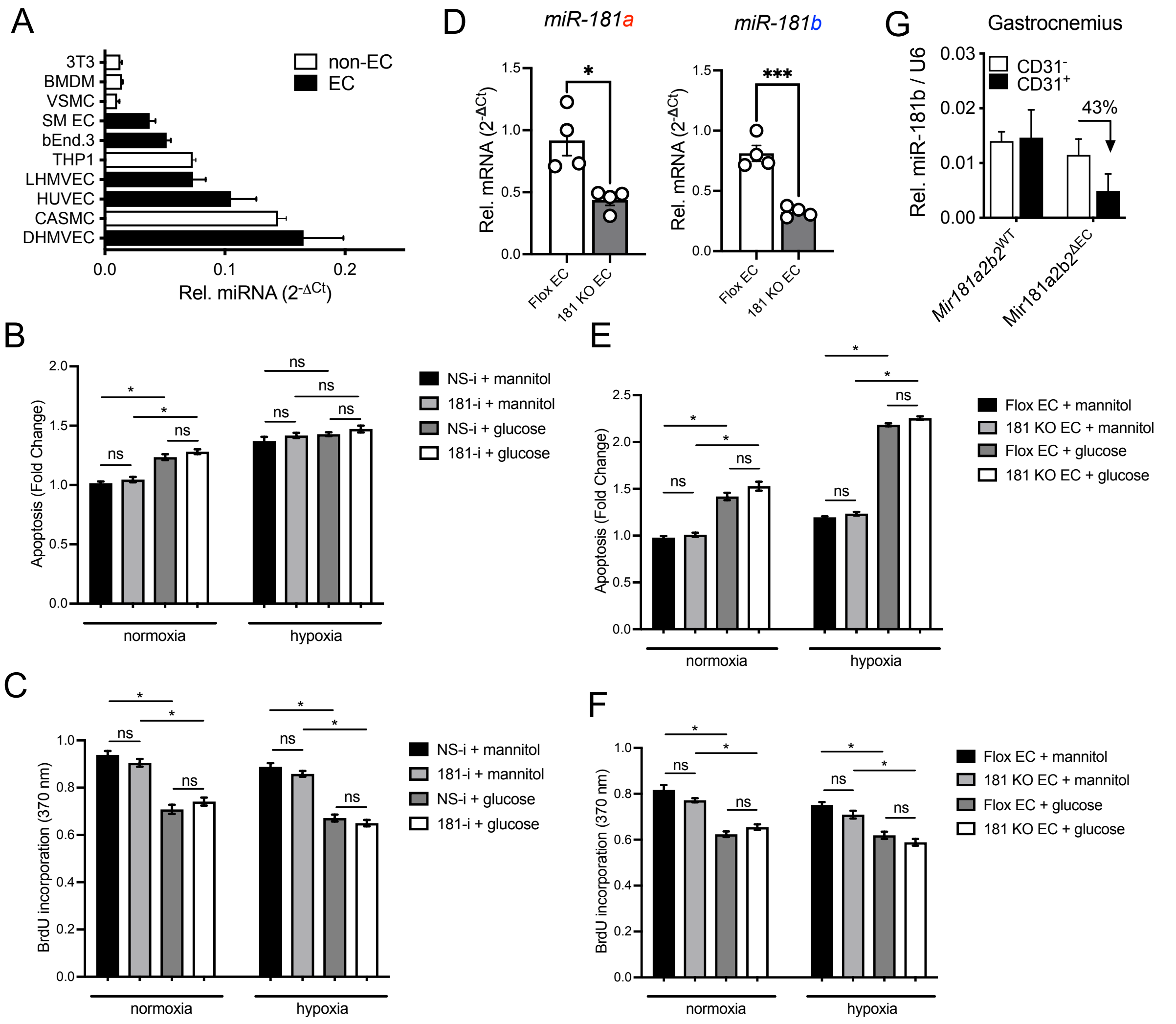

Supplemental Figure 1. Endothelial *miR-181b* kinetics and regulation of proliferation and apoptosis*.*

A. Expression of *miR-181b* normalized to *U6* in a variety of mouse (3T3 embryonic fibroblast; BMDM macrophages; VSMC vascular smooth muscle cell; SM EC skeletal muscle endothelial cell; B.End3 brain EC) and human (THP1 monocytes; LHMVEC lung microvascular EC; HUVEC umbilical vein EC; CASMC coronary artery smooth muscle cell; DHMVEC dermal microvascular EC) cell lines. HUVECs transfected with 100nM NS-i or miR-181-i and treated with ± high-glucose and ± hypoxia were subjected to **B.** apoptosis assay (n=6) and **C.** BrDU proliferation assay (n=6). **D.** Mature *miR-181a/b expression* normalized to *U6* in lung microvascular ECs from *Mir181a2b2*$^{flox/flox}$ (Flox EC) and *Mir181a2b2*$^{-/-}$ (181 KO EC) mice. Flox EC and 181 KO EC treated with ± high-glucose and ± hypoxia were subjected to **E.** apoptosis assay (n=6) and **F.** BrDU proliferation assay (n=6). **G.** CD31-positive and negative cell fraction isolated from gastrocnemius muscle from *MiR181a2b2*^{WT} and *MiR181a2b2*$^{\Delta EC}$ mice (n=4). All statistics performed with unpaired student t-test. **P* < 0.05, ****P* < 0.001.

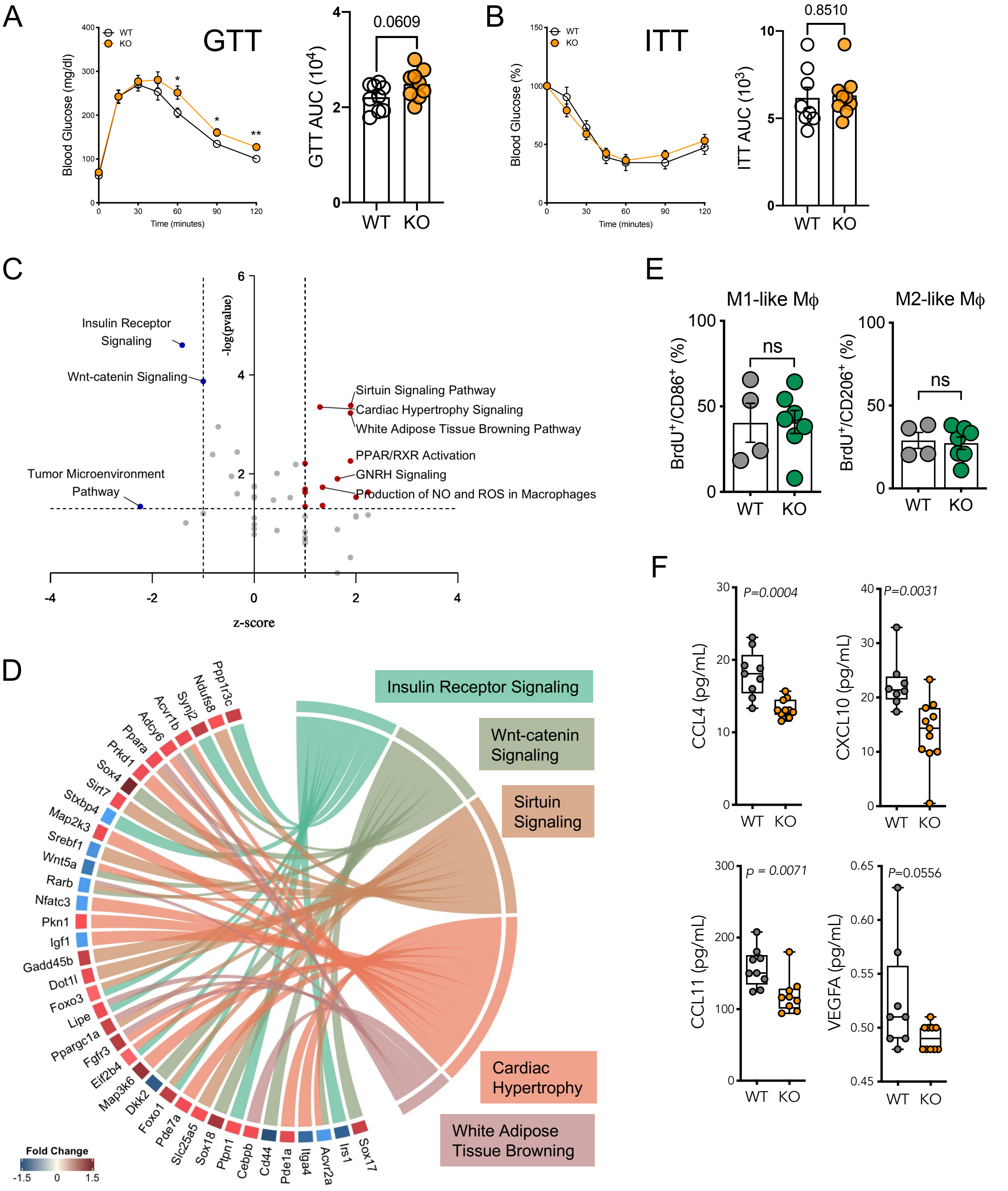

Supplemental Figure 2. Characteristics of *MiR181a2b2* KO mice on HFSC diet.

A. Glucose tolerance test (GTT) and **B.** Insulin tolerance test (ITT) performed on WT and KO mice after 4 weeks of HFSC diet, student t-test performed on area under the curve (n=9). **C.** Dysregulated pathways from transcriptome-wide analysis of ischemic gastrocnemius 3 weeks post-FAL. **F.** Chord plot highlighting top dysregulated genes contributing to dysregulated signaling pathways. **E.** BrdU measurement by flow cytometry of CD86-positive and CD206-positive macrophages in ischemic gastrocnemius 2 weeks post-FAL (n=4-7). **F.** Plasma levels of CCL4, CXCL10, CCL11, and VEGFA measured by cytokine array (n=9-11). All statistics comparing 2 groups using student t-test. *P*<0.05 is considered significant.

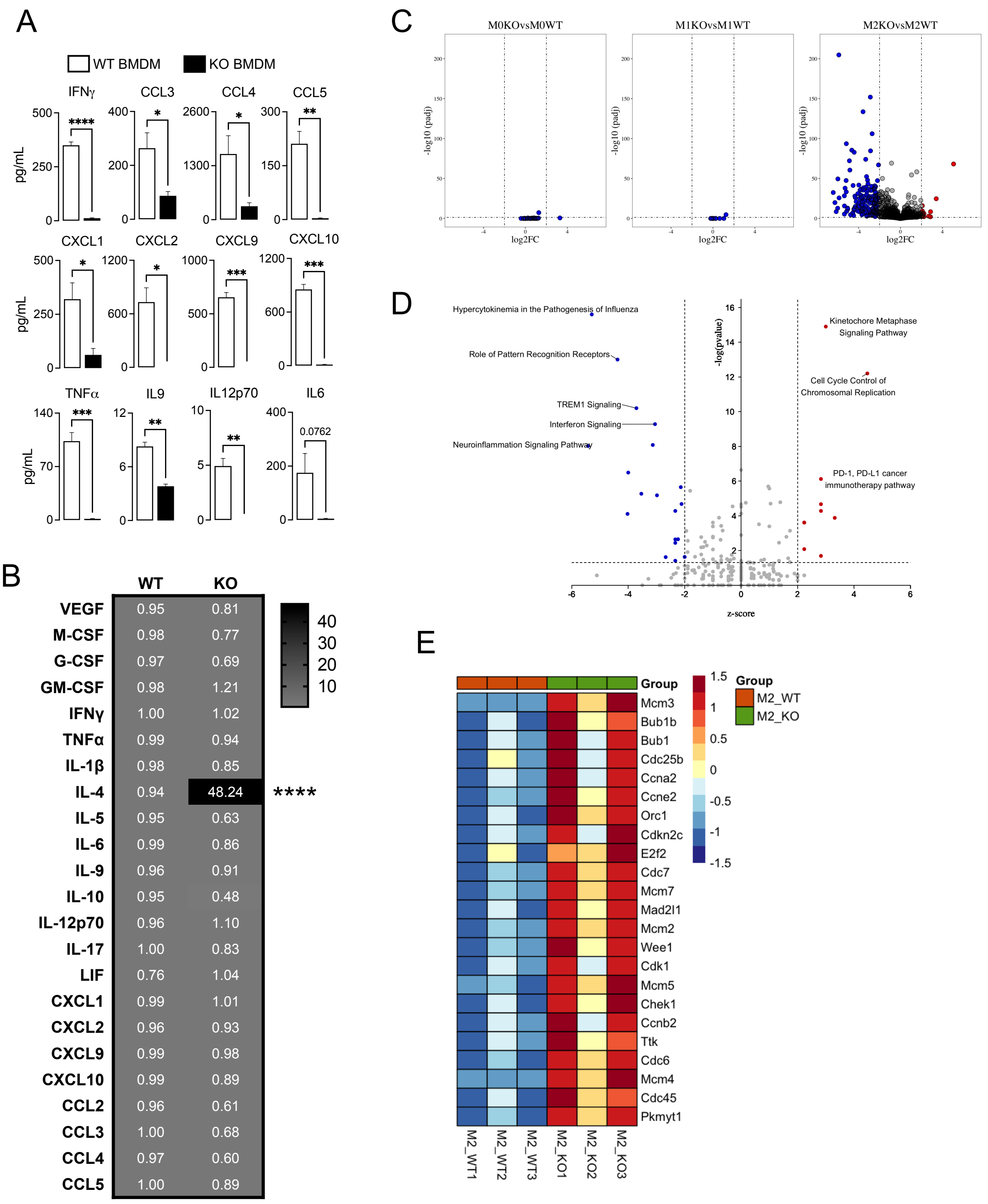

Supplemental Figure 3. Characteristics of BMDM from *MiR181a2b2* KO mice on HFSC diet.

A. Cytokine analysis from M2-like BMDM supernatant from the indicated HFSC WT or KO mice, using student t-test (n=3). **B.** Heatmap of cytokines secreted by M1-like BMDMs comparing KO to WT relative values (n=3). **C.** Volcano plots of transcriptome-wide analysis comparing KO to WT of naïve macrophages (M0), M1-like macrophages (M1), and M2-like macrophages (M2). **D.** Dysregulated pathways from transcriptome-wide analysis of M2-like macrophages. **E.** Heatmap of all genes dysregulated from the signaling pathway 'Cell Cycle Control of Chromosome Replication' in WT and KO M2-like macrophages. *$P < 0.05$, **$P < 0.01$, ***$P < 0.001$, ****$P < 0.0001$.

Supplemental Figure 4.

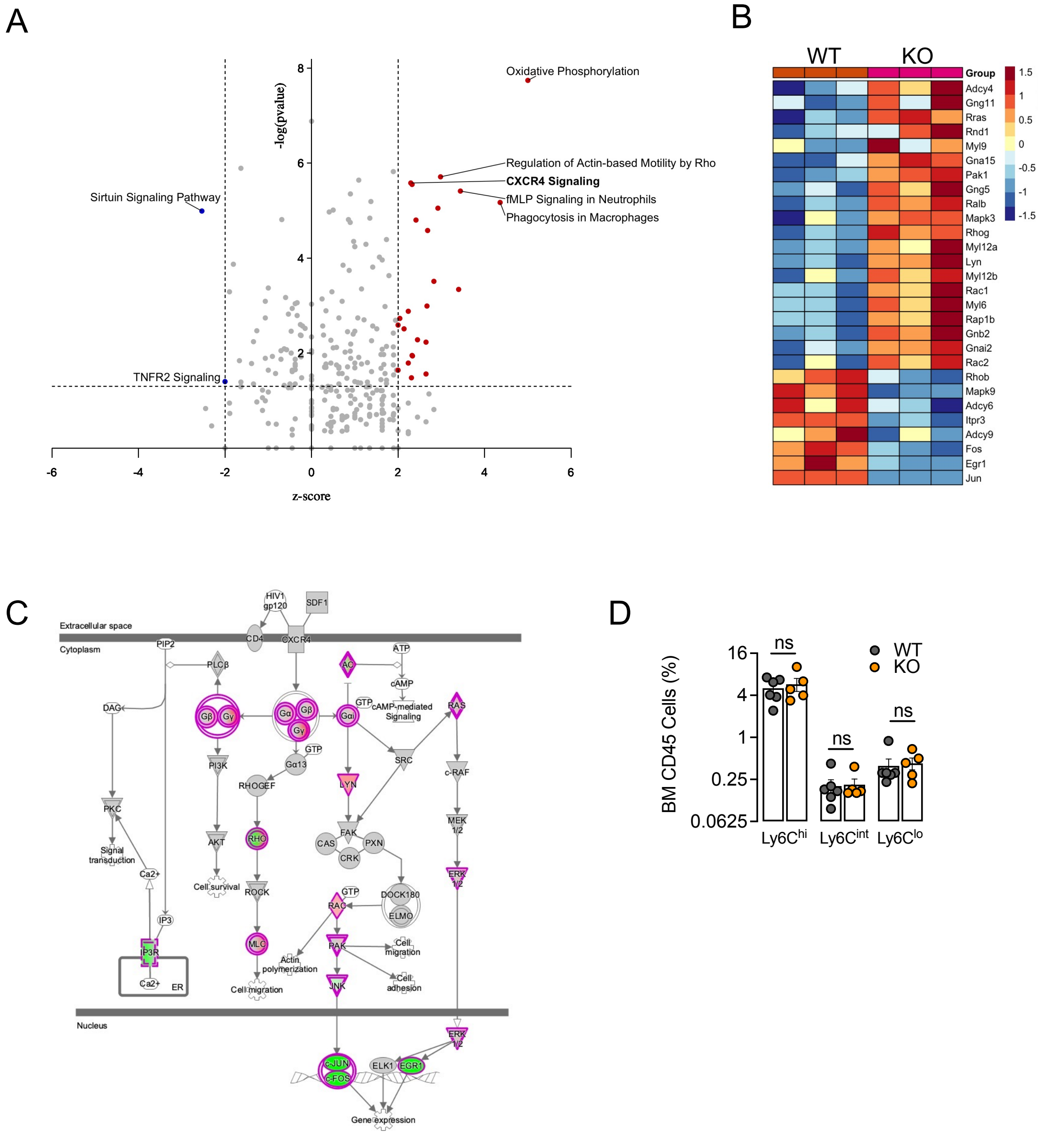

Supplemental Figure 4. Characteristics of bone marrow from *MiR181a2b2* KO mice on HFSC diet.

A. Dysregulated pathways from transcriptome-wide analysis from KO and WT BM after 6 weeks of HFSC diet. **B.** Heatmap of significantly dysregulated genes associated with CXCR4 signaling. **C.** Schema of CXCR4 signaling pathway with coloration indicating genes significantly changed when comparing KO to WT. **D.** Flow cytometry analysis within BM for monocytes with the indicated Ly6C cell surface expression, using student t-test (n=5-6). P<0.05 is considered significant.

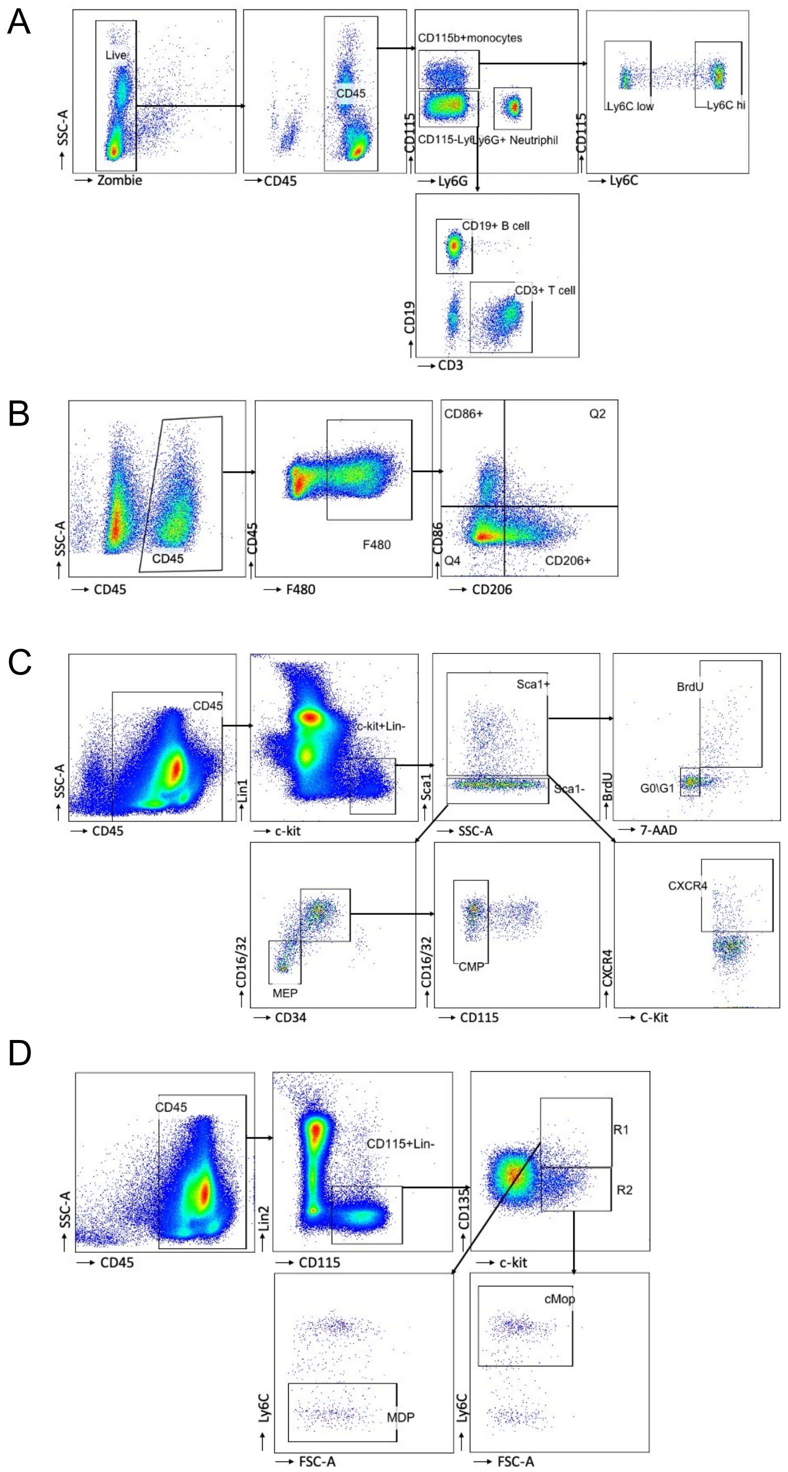

Supplemental Figure 5. Flow cytometry gating strategy.

FACS gating strategy for **A.** Peripheral blood, **B.** Ischemic gastrocnemius, **C** and **D.** Bone marrow.

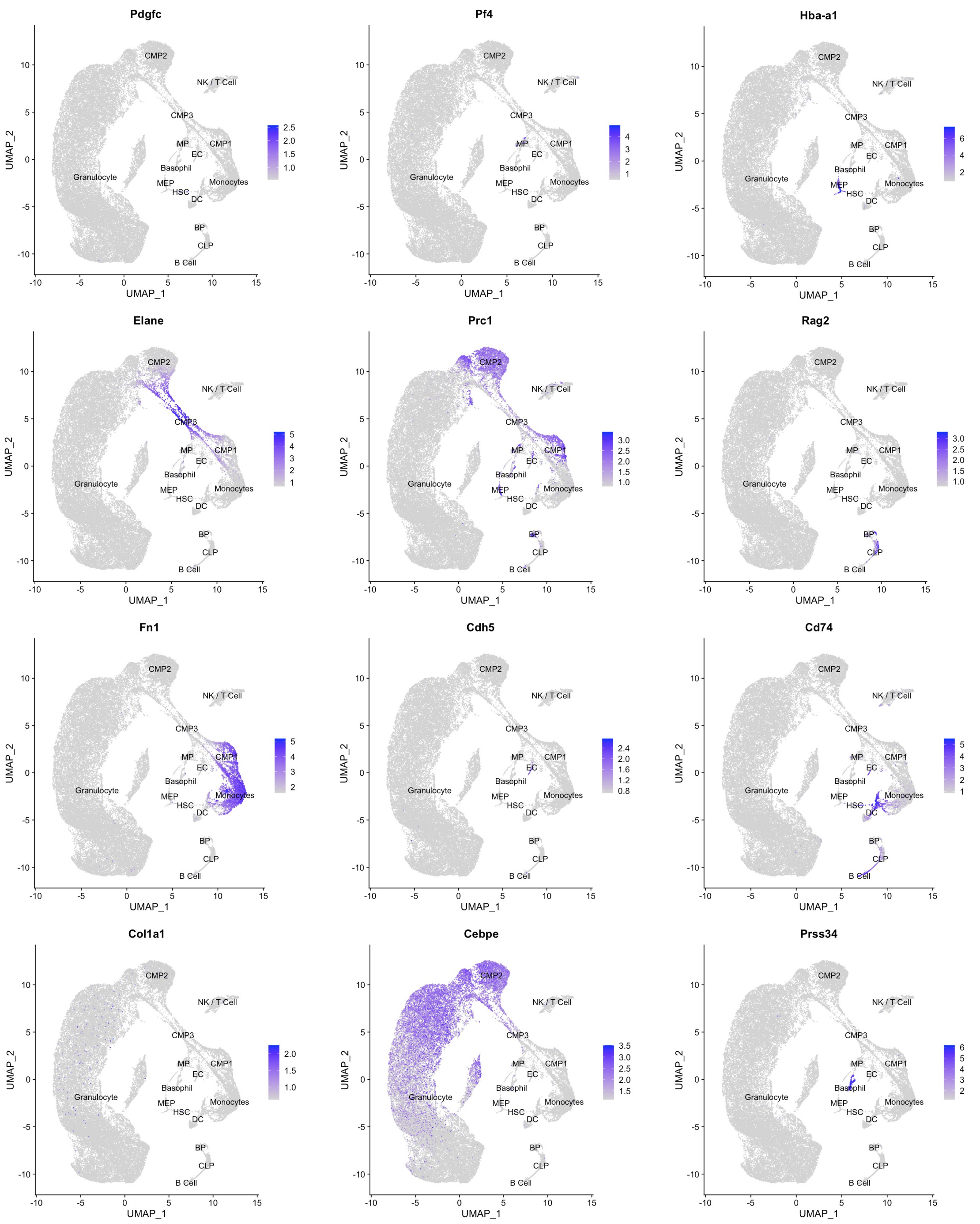

Supplemental Figure 6. Cluster specific gene expression.

UMAPs of cell specific gene expressions to classify cell clusters.

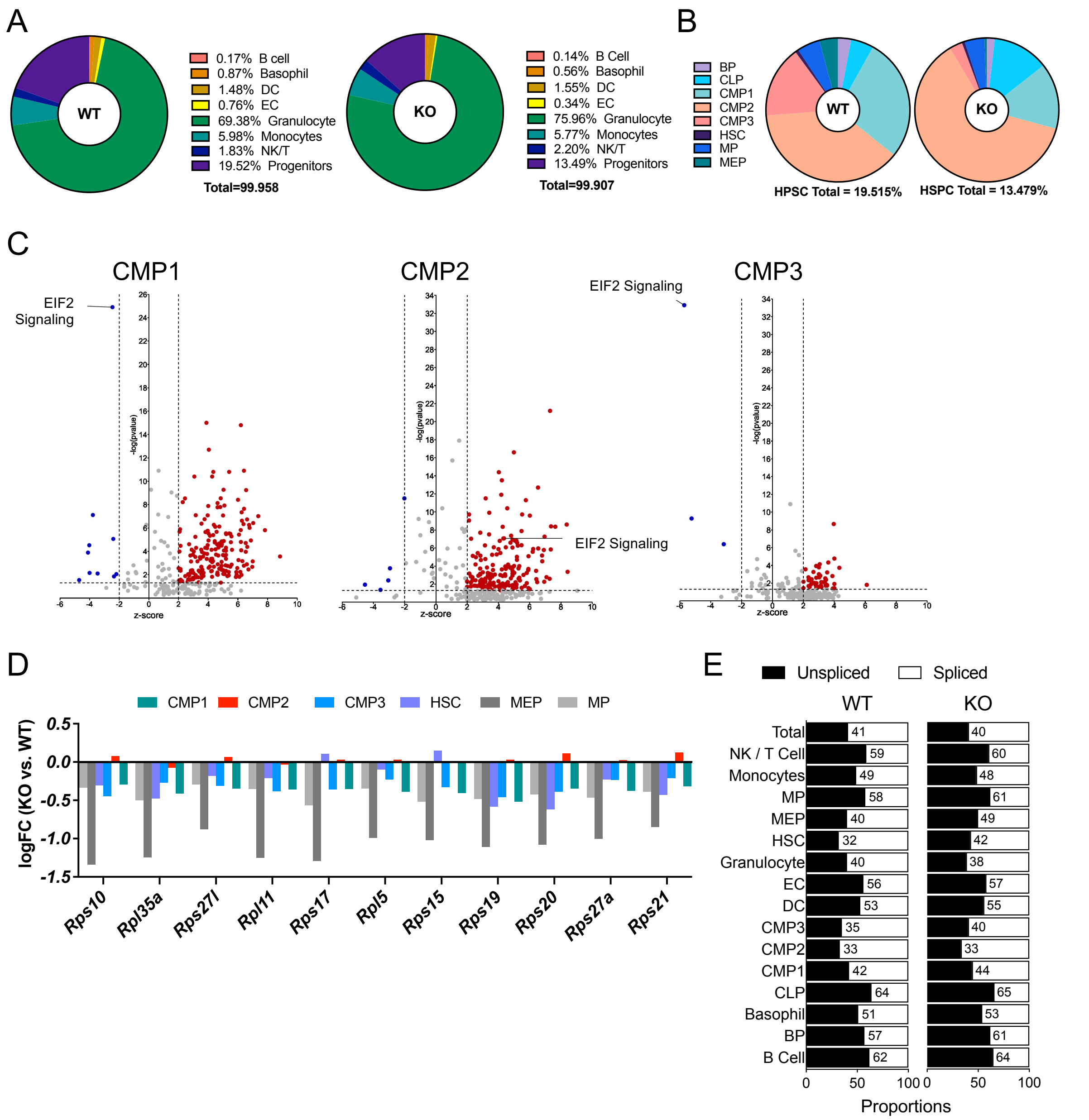

Supplemental Figure 7. scRNA-seq analysis of progenitor cell subsets.

A. Percentage of cells in BM niche of WT and KO mice. **B.** Percentage of progenitor cell subsets in BM of WT and KO mice. **C.** Dysregulated EIF2 signaling pathway from scRNA-seq analysis from KO and WT CMP1, CMP2, and CMP3 clusters. **D.** Selected dysregulated ribosome protein genes from EIF2 signaling pathway. **E.** Proportions of unspliced and spliced mRNA across the BM niche (number represent unspliced value).

Supplemental Table 1. Human subject characteristics

Characteristic	T2D FI/II (n=7)	T2D FIII/IV (n=6)
Median Age	64.46 (56-70)	63.69 (55-72)
Race	Caucasian	Caucasian
Sex	Male	Male

Supplemental Table 2. miScript miRNA primers used for RT-qPCR

**Primer Assay**	**GeneGlobe ID**	**Sequence (5' to 3')**
Hs_miR-181a_2	MS00008827	AACAUUCAACGCUGUCGGUGAGU
Hs_miR-181b_1	MS00006699	AACAUUCAUUGCUGUCGGUGGGU
Hs_miR-181c_2	MS00008841	AACAUUCAACCUGUCGGUGAGU
Hs_RNU6-2_11	MS00033740	Not available

Supplemental Table 3. Mouse primers used for RT-qPCR

**Gene**	**Forward primer（5' to 3'）**	**Reverse primer（5' to 3'）**
Cxcr4	GAAGTGGGGTCTGGAGACTAT	TTGCCGACTATGCCAGTCAAG
Cxcl12	TGCATCAGTGACGGTAAACCA	TTCTTCAGCCGTGCAACAATC
Hprt	TCAGTCAACGGGGGACATAAA	GGGGCTGTACTGCTTAACCAG

Supplemental Table 4. Antibodies used for flow cytometry analysis

Antibody	Fluorochrome	Catalog	Trade name
anti-mouse CD45	BUV563	612924	BD
anti-mouse CD45	PE-Cyanine7	103114	BioLegend
anti-mouse CD115	BV711	135515	BioLegend
anti-mouse CD115	BV605	743640	BD
anti-mouse CD48	BC605	103441	BioLegend
anti-mouse CXCR4	BV421	562738	BD
anti-mouse CXCR4	FITC	551967	BD
anti-mouse SCA-1	PE-Cyanine7	25-5981-82	Invitrogen
anti-mouse CD117	APC-Cy7	105838	BioLegend
anti-mouse CD150	PerCP-Cy5.5	46-1502-82	Thermo Fisher
anti-mouse CD16/32	Alexa 700	565502	BD
anti-mouse CD34	APC	560230	BD
anti-mouse Ly6C	FITC	128006	BioLegend
anti-mouse F4/80	PE-Cyanine7	123114	BioLegend
anti-mouse F4/80	APC	123116	BioLegend
anti-mouse CD135	BV421	562898	BD
anti-mouse CD11b	PerCP-Cy5.5	101228	BioLegend
anti-mouse CD11b	PE	101208	BioLegend
anti-mouse Ly6G	APC	127614	BioLegend
anti-mouse CD3	Pacific blue	103114	BioLegend
anti-mouse CD3	PE	100206	BioLegend
anti-mouse CD4	PerCP-Cy5.5	100434	BioLegend
anti-mouse CD86	BV605	563350	BD
anti-mouse CD206	PerCP-Cy5.5	141716	BioLegend
anti-mouse B220	PE	103208	BioLegend
anti-mouse Ly6G	PE	127608	BioLegend
anti-mouse CD11c	PE	117308	BioLegend
anti-mouse NK1.1	PE	108708	BioLegend
anti-mouse Ter119	PE	116208	BioLegend
anti-mouse CD127	PE	135010	BioLegend
anti-mouse CD90	PE	105308	BioLegend
anti-mouse CD309	Alexa 488	136408	Biolegend
anti-mouse CD133	PE-Cyanine7	141210	Biolegend

Supplemental Table 5. Cell cluster naming

Cell Cluster	Enriched gene(s)
Hematopoietic Stem Cell	*Pdgfc, Ghr, Bgn, Prnp, Apoe, Mfge8*
Common Myeloid Progenitor 1 & 2	*Ccna2, Prc1, Kif11*
Common Myeloid Progenitor 3	*Elane, Ctsg, Mpo*
Common Lymphoid Progenitor	*Rag2, Cnn3, Rasal1*
Megakaryote-Erythroid Progenitor	*Hba-a1, Smim1, Gata1, Gypa*
Megakaryote Progenitor	*Pf4, Itg2b, Mef2c*
Monocytes	*Fn1, Ccr2*
Dendritic Cells	*Cd74*
Neutrophils / Eosinophils	*Cebpe*
Basophils	*Prss34, Runx1*
Pre-B Cells	*Vpreb1, Blnk*
B Cells	*Cd79a, Cd79b*
Natural Killer / T Cells	*Ccl5, Cd3*
Endothelial Cells	*Cd31*
Osteoblasts	*Col1a1*

Supplemental Table 6. Relative expression of ribosome protein genes between KO and WT BM progenitors.

	CMP1		CMP2		CMP3		HSC		MEP		MP	
	FDR	logFC	FDR	logFC	FDR	logFC	pvalue	logFC	FDR	logFC	FDR	logFC
Rps10	7.32E-22	-0.29	0.0102	0.0757	3.04E-12	-0.444	0.202	-0.304	0.0872	-1.34	1.84E-06	-0.334
Rpl35a	4.64E-43	-0.41	0.00752	-0.0719	1.07E-05	-0.267	0.243	-0.476	0.0872	-1.24	6.29E-12	-0.501
Rps27l	1.93E-18	-0.346	0.0156	0.0652	0.00196	-0.311	0.359	-0.177	0.0872	-0.88	0.0111	-0.292
Rpl11	4.13E-23	-0.356	0.105	-0.0337	1.77E-07	-0.379	0.76	-0.211	0.0872	-1.25	3.26E-05	-0.353
Rps17	2.58E-25	-0.353	0.474	0.0291	8.03E-08	-0.357	0.598	0.106	0.0872	-1.29	3.00E-11	-0.562
Rpl5	7.28E-30	-0.389	0.894	0.0299	0.0019	-0.228	0.616	-0.0995	0.192	-0.988	8.31E-07	-0.343
Rps15	1.83E-35	-0.404	0.981	-0.00501	3.78E-08	-0.33	0.452	0.147	0.308	-1.02	4.33E-12	-0.519
Rps19	1.88E-52	-0.518	0.0401	0.0321	7.65E-12	-0.457	0.0288	-0.585	0.339	-1.11	1.50E-10	-0.483
Rps20	7.86E-24	-0.345	6.82E-05	0.111	1.77E-07	-0.385	0.107	-0.619	0.404	-1.08	7.60E-09	-0.423
Rps27a	3.40E-30	-0.377	0.484	0.0227	0.000691	-0.232	0.573	-0.229	0.474	-1	2.32E-10	-0.462
Rps21	1.32E-20	-0.314	7.51E-05	0.121	0.0073	-0.206	0.452	-0.426	0.63	-0.846	5.74E-09	-0.386
